# Metabolic Linkage and Correlations to Storage Capacity in Erythrocytes from Glucose 6-Phosphate Dehydrogenase-Deficient Donors

**DOI:** 10.3389/fmed.2017.00248

**Published:** 2018-01-11

**Authors:** Julie A. Reisz, Vassilis L. Tzounakas, Travis Nemkov, Artemis I. Voulgaridou, Issidora S. Papassideri, Anastasios G. Kriebardis, Angelo D’Alessandro, Marianna H. Antonelou

**Affiliations:** ^1^Department of Biochemistry and Molecular Genetics, School of Medicine, University of Colorado, Aurora, CO, United States; ^2^Department of Biology, School of Science, National and Kapodistrian University of Athens, Athens, Greece; ^3^“Apostle Paul” Educational Institution, Thessaloniki, Greece; ^4^Department of Medical Laboratories, Faculty of Health and Caring Professions, Technological and Educational Institute of Athens, Athens, Greece

**Keywords:** glucose 6-phosphate dehydrogenase deficiency, transfusion medicine, red blood cell storage lesion, donor variation, mass spectrometry, metabolomics, interactome

## Abstract

**Objective:**

In glucose 6-phosphate dehydrogenase (G6PD) deficiency, decreased NADPH regeneration in the pentose phosphate pathway and subnormal levels of reduced glutathione result in insufficient antioxidant defense, increased susceptibility of red blood cells (RBCs) to oxidative stress, and acute hemolysis following exposure to pro-oxidant drugs and infections. Despite the fact that redox disequilibrium is a prominent feature of RBC storage lesion, it has been reported that the G6PD-deficient RBCs store well, at least in respect to energy metabolism, but their overall metabolic phenotypes and molecular linkages to the storability profile are scarcely investigated.

**Methods:**

We performed UHPLC-MS metabolomics analyses of weekly sampled RBC concentrates from G6PD sufficient and deficient donors, stored in citrate phosphate dextrose/saline adenine glucose mannitol from day 0 to storage day 42, followed by statistical and bioinformatics integration of the data.

**Results:**

Other than previously reported alterations in glycolysis, metabolomics analyses revealed bioactive lipids, free fatty acids, bile acids, amino acids, and purines as top variables discriminating RBC concentrates for G6PD-deficient donors. Two-way ANOVA showed significant changes in the storage-dependent variation in fumarate, one-carbon, and sulfur metabolism, glutathione homeostasis, and antioxidant defense (including urate) components in G6PD-deficient vs. sufficient donors. The levels of free fatty acids and their oxidized derivatives, as well as those of membrane-associated plasticizers were significantly lower in G6PD-deficient units in comparison to controls. By using the strongest correlations between *in vivo* and *ex vivo* metabolic and physiological parameters, consecutively present throughout the storage period, several interactomes were produced that revealed an interesting interplay between redox, energy, and hemolysis variables, which may be further associated with donor-specific differences in the post-transfusion performance of G6PD-deficient RBCs.

**Conclusion:**

The metabolic phenotypes of G6PD-deficient donors recapitulate the basic storage lesion profile that leads to loss of metabolic linkage and rewiring. Donor-related issues affect the storability of RBCs even in the narrow context of this donor subgroup in a way likely relevant to transfusion medicine.

## Introduction

Routine storage of packed red blood cells (RBCs) in the blood bank is a logistic necessity that makes ~110 millions of units available for life-saving transfusions to millions of recipients worldwide every year. Storage in the blood bank is associated with the progressive accumulation of a series of biochemical and morphological alterations to RBCs collectively referred to as the storage lesion (1, 2). Deranged metabolic homeostasis of stored RBCs is a heritable trait, i.e., it is affected—like hemolysis—by the donor’s genetic background (3–5). The metabolic storage lesion can be cursorily summarized in two main components, i.e., decreased energy metabolism and increased oxidative stress (6–10). Despite laboratory observations suggesting that old blood may be associated with poorer transfusion outcomes, reassuring evidence from randomized clinical trials has been generated to support the overall safety and efficacy of current transfusion practices (11). The apparent disconnect on the age of blood issue between laboratory observations and randomized clinical trials is in part reconciled by the appreciation of the many confounding factors affecting the interpretation of prospective clinical trials and limited size of cohorts tested in most basic science/laboratory studies. Introduction of high-throughput omics technologies and combination of omics results with functional outcomes (12) has fostered a new era in the field of transfusion medicine where the focus has been shifted from the final product to the donor (13–15) and the intrinsic variability across the donor population. Animal studies further strengthened this conclusion, reporting that while not all (mouse strain) donor RBCs store similarly (16), transfusion of RBCs from different (mouse strain) donors may result in a “good apple/bad apple” effect (17), further increasing the complexity of the donor/recipient system and increasing the noise of clinical studies where exclusively and consistently young or old blood is hardly ever transfused to the same recipient (18). In humans, factors such as donor age, ethnicity, and gender ultimately affect RBC storability (influencing parameters such as hemolysis or oxidative stress-induced hemolysis) (12). Gender in particular may be an underestimated confounder (19).

Glucose 6-phosphate dehydrogenase (G6PD) deficiency is an X-linked (20) recessive inborn error of metabolism that affects ~400 million individuals worldwide and results in impaired antioxidant capacity. Indeed, carriers of G6PD-deficient traits are characterized by a reduced capacity to generate antioxidant equivalents (i.e., NADPH) through the pentose phosphate pathway (PPP), which in turn results in an increased susceptibility to hemolysis. As oxidative stress has been considered an etiological contributor to the RBC storage lesion, it has been anticipated that RBCs from G6PD-deficient donors may suffer from exacerbated alterations during storage in the blood bank (21). While clinical evidence on the issue is still missing, preliminary omics studies have revealed that RBCs from G6PD-deficient donors unexpectedly better preserve energy homeostasis and morphology during storage in the blood bank though they are increasingly more susceptible to temperature and oxidative stress-induced hemolysis than stored RBCs from G6PD sufficient donors (22, 23). In that preliminary study, we focused specifically on glycolysis and the PPP. However, Tang and colleagues (24) recently showed that RBCs from G6PD-deficient donors challenged with pro-oxidant stimuli such as diamide are characterized by a wide series of alterations, including alterations of purine homeostasis which in turn result in activation of AMP protein kinase. Other pathways, such as fatty acid metabolism, are significant correlates to post-transfusion recoveries in mouse models (25, 26). In this study, we expand on our previous observation on the metabolic phenotypes of RBCs from G6PD-deficient vs. sufficient donors. Results are correlated to physiological measurements of potential clinical impact, such as extracellular potassium, oxidative lesions, RBC fragility, and susceptibility to hemolysis *in situ* or at post-storage mimicking conditions (e.g., incubation at 37°C).

## Materials and Methods

### Subjects, Blood Collection, and Processing

Six male, 22–30 years old G6PD-deficient (G6PD^−^, Mediterranean variant, <10% residual activity of the enzyme) and three gender- and age-matched G6PD-normal (G6PD^+^) regular blood donors were recruited. Venous blood was collected into EDTA or citrate vacutainers just before blood donation and preparation of packed RBCs. RBC storage quality was evaluated in citrate phosphate dextrose (CPD)/saline adenine glucose mannitol (SAGM) log4 leukofiltered units (Haemonetics Corp., MA, USA) stored for 42 days at 4–6°C. Samples were collected aseptically at weekly intervals of the storage period (days 7, 14, 21, 28, 35, and 42). The study was approved by the Ethics Committee of the Department of Biology, School of Science, NKUA. Investigations were carried out upon signing of written consent, in accordance with the principles of the Declaration of Helsinki.

### Hematological, Biochemical, and Physiological Measurements

Pre-donation blood and RBC concentrates of G6PD-deficient donors were further evaluated for almost 45 hematological, biochemical, and physiological parameters before and throughout the storage period in CPD/SAGM, as described in the previously published study (22, 23). Shortly, Hb concentration and RBC indexes (RBC and reticulocyte counts, hematocrit, mean corpuscular volume, mean corpuscular Hb, mean corpuscular Hb concentration, and RBC distribution width) were measured using the Sysmex K-4500 automatic blood cell counter (Roche), while serum biochemical analysis (triglycerides, cholesterol, low density lipoproteins, high density lipoproteins, iron, ferritin, total billirubin, uric acid, aspartate transaminase, alanine aminotransferase, potassium, and sodium) was performed using the analyzers Hitachi 902, 9180 and Elecsys Systems Analyzer (Roche). Levels of glycated Hb (HbA1c) and G6PD activity were measured in fresh blood and in packed RBCs on the last day of storage. Levels of extracellular (free) Hb, total or uric acid-dependent/independent antioxidant activities, total and RBC-derived microparticles (MPs), and MP-associated pro-coagulant activity were evaluated in plasma/supernatant by standard biochemical assays, flow cytometry, or ELISA approaches. Fresh and stored RBCs were finally evaluated for shape modifications (scanning electron microscopy), osmotic and mechanical fragility, membrane protein carbonylation, and accumulation of intracellular reactive oxygen species (ROS) and calcium. Measurements of RBC fragilities and ROS accumulation were performed before and after 24 h incubation at 37°C, while ROS accumulation was estimated before and after treatment with the oxidative agents diamide (dROS) and *tert*-butyl hydroperoxide (tBHP, tROS). All measurements were run in triplicate.

### Metabolomics Analyses

Metabolomics analyses were performed as previously reported (22). Briefly, 100 µL of stored RBCs were collected on a weekly basis and extracted at 1:6 dilutions in methanol:acetonitrile:water (5:3:2), vortexed, and centrifuged to pellet proteins, prior to analysis by UHPLC-MS (Ultimate 3000 RSLC-Q Exactive, Thermo Fisher). Sample extracts (10 µL) were loaded onto a Kinetex XB-C18 column (150 mm × 2.1 mm × 1.7 µm—Phenomenex, Torrance, CA, USA). A 9-min gradient from 5 to 95% B (phase A: water + 0.1% formic acid and B: acetonitrile + 0.1% formic acid) eluted metabolites into a Q Exactive system (Thermo, Bremen, Germany), scanning in full MS mode (3 min method) or performing acquisition independent fragmentation (MS/MS analysis—9 min method) at 70,000 resolution in the 60–900 *m*/*z* range, 4 kV spray voltage, 15 sheath gas, and 5 auxiliary gas, operated in negative and then positive ion mode (separate runs). Metabolite assignment was performed against an in house standard library, as reported (27), through the freely available software Maven (Princeton University, USA) (28). No data pre-processing (neither normalization nor log-transformation) was performed. In our previous study (22, 23), only glycolysis, ribose phosphate, glutathione, and NADH/NAD+ ratios were reported. Here, we expanded the analysis to amino acids, lipids, purines, and other metabolites, as extensively reported in Table S1 in Supplementary Material.

### Statistics

For statistical analysis, the Statistical Package for Social Sciences (SPSS, IBM) was used. Correlations between parameters were evaluated by the Pearson’s and Spearman’s tests after checking out the variables for normal distribution profile (by using the Shapiro–Wilk test) and presence of outliers. Briefly, in the absence of normal distribution, Spearman test was performed. In addition, and since Pearson’s test is sensitive to extreme outliers, in the presence of such an outlier the value was excluded and the analysis was performed again, to minimize the possibility of false results likely associated with the small size of the cohort. If the outcome of the subsequent Pearson analysis was not modified compared to the first one, the outlier was included back to the cohort. If not, Spearman analysis was preferred. Outliers (any measurement outside the range of mean ± 2 × SD) were identified by using both the Shapiro–Wilk test and detrended normal Q–Q plots. Significance was accepted at a *p* value of less than 0.01.

### Network Analysis

All hematological, biochemical, omics, and physiological parameters collected from G6PD-deficient donors (for abbreviations, see Table S2 in Supplementary Material) were used for the construction of biological networks connecting variables of fresh donor’s blood (*in vivo* state) with those of packed RBCs (*ex vivo* state) by significant and repeated correlations that existed throughout (namely, at every time point of) the storage period (with the exception of the G6PD activity and percentage of HbA1c, for which only end-of-storage measurements were available). The reasoning behind selection of correlations that were repeatedly evident at all time points of storage (namely, fresh blood vs. 7th and 14th and so on until the 42nd day) was to find out sound links between variables regardless of storage duration and in parallel, to minimize the false discovery rate that is intrinsically connected to any small sized sample. To increase the confidence level, the outputs of that analysis were further analyzed by a Bonferroni-like correction for multiple comparisons. The multiply checked and thus, most probably true, correlations were topologically represented in undirected biological networks by using Cytoscape version 3.2.0 application, as previously described (15). The length of each edge was inversely proportional to the *r* value (the shortest the edge, the higher *r* value).

## Results and Discussion

### Metabolic Phenotypes of G6PD-Deficient Donors Recapitulate the Storage Lesion Observed in G6PD Sufficient Donors

**Figure 1:**
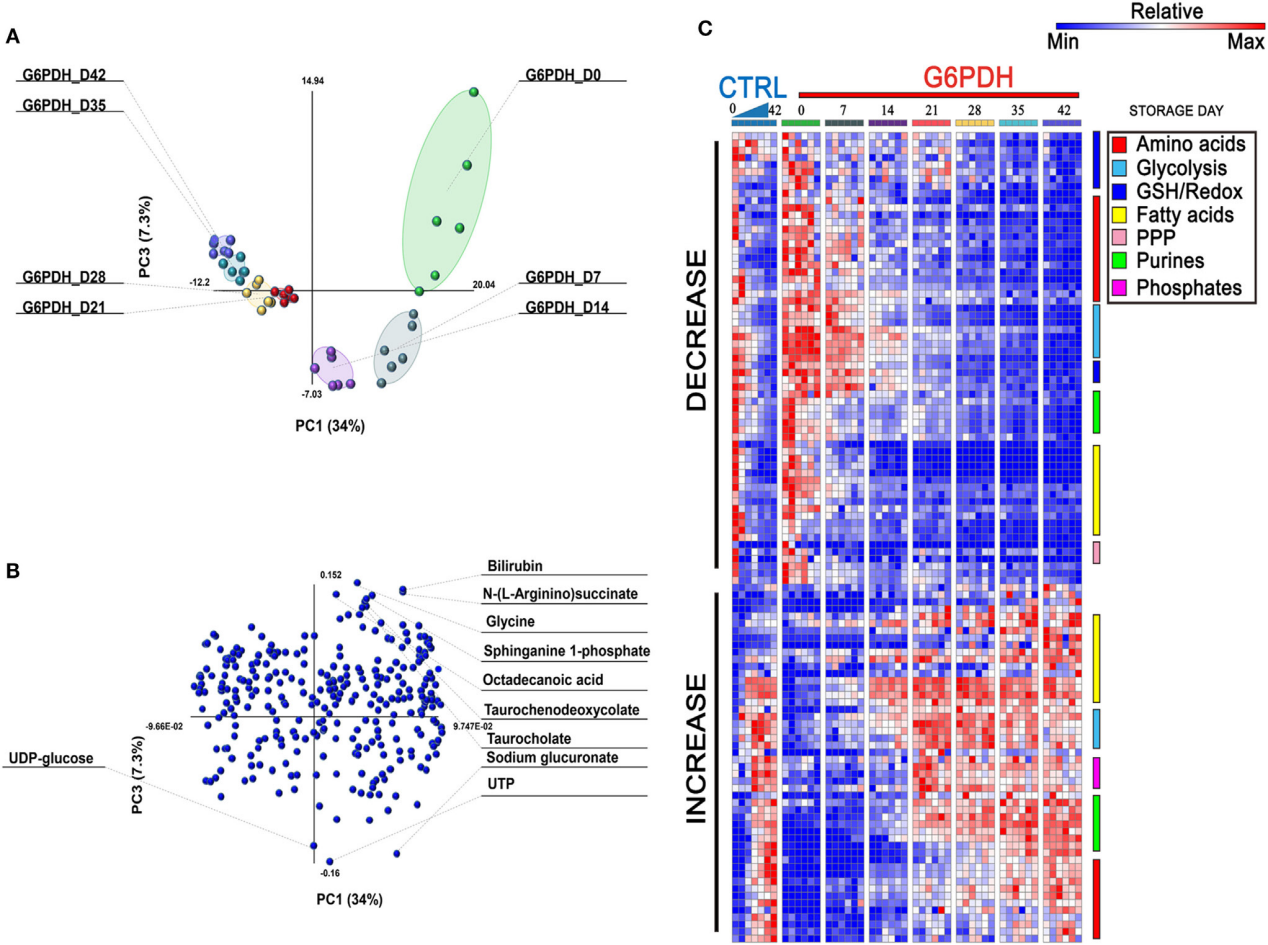
Multivariate analysis of metabolic phenotypes of stored red blood cells (RBCs) from glucose 6-phosphate dehydrogenase (G6PD)-deficient donors. Partial least square discriminant analysis [PLS-DA—panel **(A)**] revealed a U-shaped distribution of packed RBC samples from G6PD-deficient donors over storage in the blood bank, consistent with previous reports from Palsson’s lab (29, 30) and our group (9, 31). A loading plot for most significant variables informing the PLS-DA discrimination is shown in panel **(B)**, and includes bioactive lipids and bile acids. In panel **(C)**, a heat map of time course metabolic changes in RBCs from G6PD sufficient (median) or deficient (*n* = 6) donors, divided by pathway as specified by color codes in the top right corner of the panel.

Overall, a total of 293 metabolites were monitored in this study, as extensively reported in Table S1 in Supplementary Material. Recently, Palsson’s group (29, 30) recognized three stages identifying the metabolic age of blood (18). Multivariate analysis of metabolomics data from RBC concentrates stored in different additives (29–31) results in a U-shaped graph which is indicative of three time-dependent metabolic phases as RBCs age during storage. Consistently, Paglia et al. have reported that transition from phase 2 to phase 3 occurs after storage day 18 (29). Here, multivariate analysis of metabolomics data from SAGM-stored RBCs from G6PD-deficient donors suggests that such transition may occur earlier in this population (storage day 14—Figure 1A), though greater temporal resolution in the 10–18 storage day range would be necessary to further support this conclusion. Overall, the top variables discriminating RBC concentrates for G6PD-deficient donors include bioactive lipids and free fatty acids, bile acids, glycolytic metabolites, purines, and amino acids, as reported in the loading plot and heat maps in Figures 1B,C, respectively. A vectorial version of the heat map with hierarchical clustering is provided as Figure S1 in Supplementary Material.

### G6PD-Deficient Donors Are Characterized by Alterations in One-Carbon Metabolism, Glutathione/Urate Homeostasis, and Fatty Acid Metabolism Compared to G6PD Sufficient Donors

**Figure 2:**
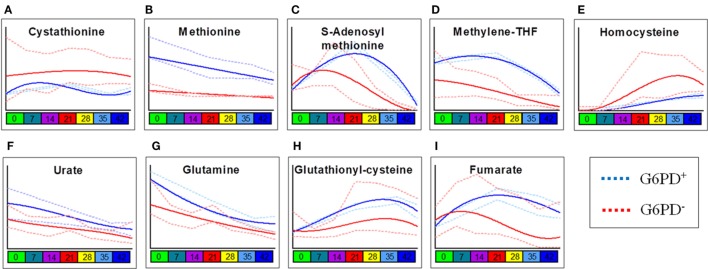
Most significant metabolic differences between glucose 6-phosphate dehydrogenase (G6PD) sufficient and deficient red blood cells (RBCs). Panels indicate alterations to sulfur/one-carbon metabolism **(A–E)**, purine oxidation **(F)**, glutathione homeostasis **(G,H)**, and carboxylates **(I)** in G6PD sufficient (blue line) and deficient (red line) RBCs during storage in the blood bank (median + ranges are shown in light blue or red for both G6PD sufficient and deficient groups, respectively).

Two-way ANOVA comparing storage-dependent trends in G6PD-deficient vs. sufficient donors revealed significant changes in metabolites involved in one-carbon and sulfur metabolism (including cystathionine, methionine, S-adenosyl-l-methionine, methylenetetrahydrofolate, and homocysteine—Figures 2A–E), metabolites involved in glutathione homeostasis and antioxidant defenses (urate, glutamine, glutathionylcysteine—Figures 2F–H), and the carboxylic acid fumarate (Figure 2I), all significantly lower in the G6PD-deficient group except for cystathionine and homocysteine.

**Figure 3:**
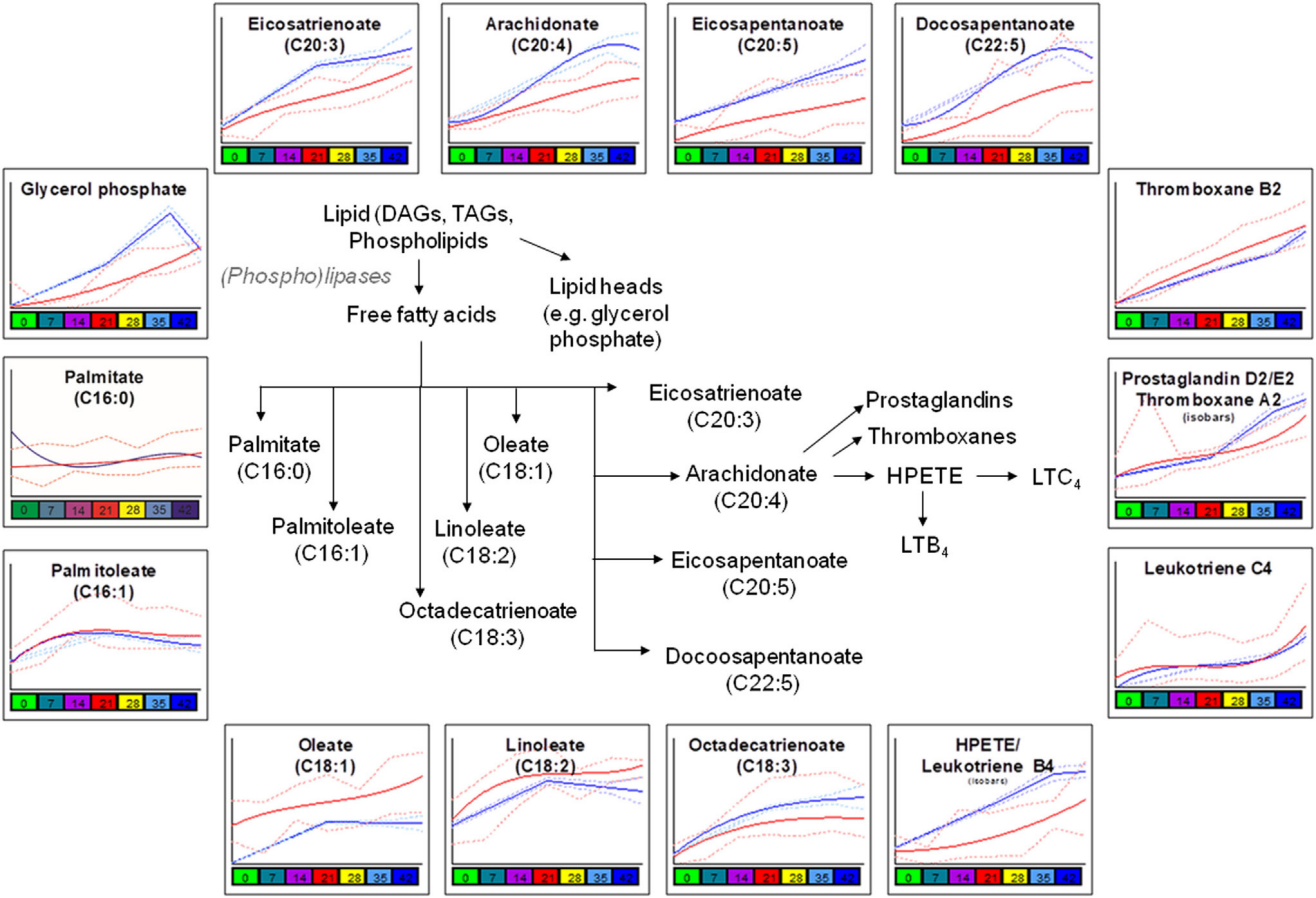
Significant differences in fatty acid metabolism and oxidation between glucose 6-phosphate dehydrogenase (G6PD) sufficient (blue line) and deficient (red line) red blood cells during storage in the blood bank (median + ranges are shown in light blue or red for both G6PD sufficient and deficient groups, respectively).

Consistent with a better preserved morphology (22) and a trend of decreased storage vesiculation degree (23), the levels of free fatty acids and oxidized derivatives (e.g., HPETE/LTB4 or isobaric isomers) were significantly lower in G6PD-deficient donors in comparison to control RBCs, with the exception of oleate and linoleate (Figure 3). Previous proteomics analyses revealed increased oxidation and stress markers accumulation but also increased levels of antioxidant enzymes in the plasma membrane and the extracellular vesicles released by the stored G6PD^−^ RBCs (22). Of note, the levels of several polyunsaturated fatty acids, including the linoleate, were found to be both heritable and associated with ATP levels in AS-3 stored RBCs (4), while in this study, the linoleate concentration in G6PD^−^ donors at donation time had a strong negative correlation with the 2,3-biphosphoglycerate (2,3-BPG) levels in stored RBCs (see below). The different concentration of oleate in fresh and stored G6PD^−^ blood compared to control blood may be associated with a different rate of incorporation into phosphatidylcholine that significantly decreases during storage (32).

**Figure 4:**
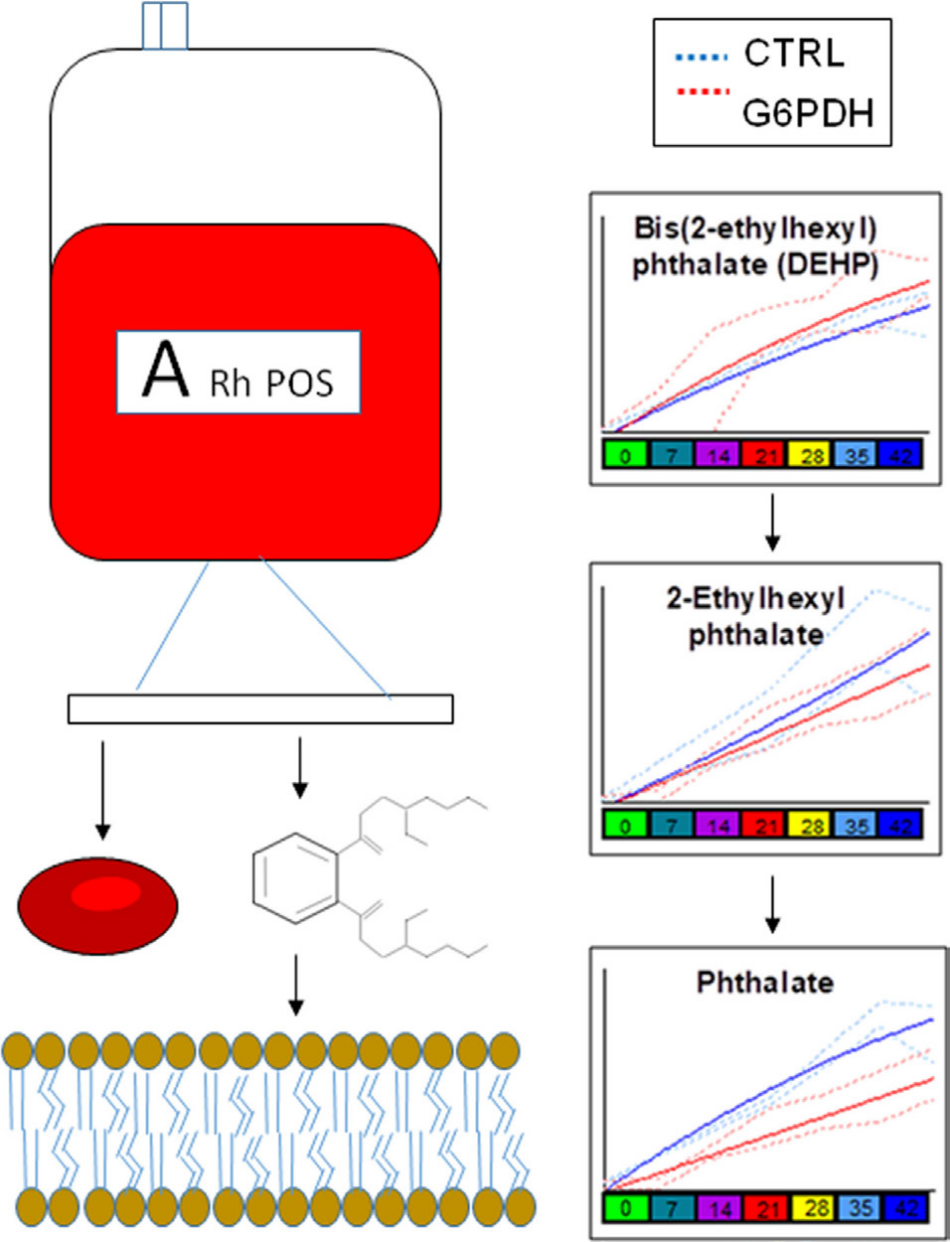
Different levels of phthalate plasticizers and breakdown products are observed in red blood cells from glucose 6-phosphate dehydrogenase (G6PD) sufficient (blue line) and deficient (red line) during storage in the blood bank (median + ranges are shown in light blue or red for both G6PD sufficient and deficient groups, respectively).

Similarly, lower levels of plasticizers monoethyl-hexylphthalate and phthalate were detected in RBC concentrates from G6PD-deficient donors (Figure 4). Approximately 28% of the available bis(2-ethylhexyl) phthalate (DEHP) is taken up by stored RBCs where it exerts a protective effect on membrane stability and flexibility similar to that of mannitol (33). Since NADPH is indispensable for the synthesis of fatty acids and cholesterol, the RBC membrane in G6PD deficiency of Mediterranean type is characterized by increased fluidity and decreased cholesterol-to-phospholipid ratio (34). Considering that RBCs from both donor cohorts were processed and stored through comparable manufacturing processes, and that the DEHP levels in G6PD^−^ units were equal to the control levels, it is plausible to speculate that the lipid remodeling in G6PD deficiency may favor incorporation of DEHP in the membrane, preventing thus its hydrolysis to MEHP and phthalate. This kind of protective effect is consistent with the previously reported trend of stored G6PD^−^ RBCs to reduced mechanical fragility compared to that of control RBCs at body temperature (23).

### Loss of Metabolic Linkage and Metabolic Rewiring in G6PD-Deficient Donors

**Figure 5:**
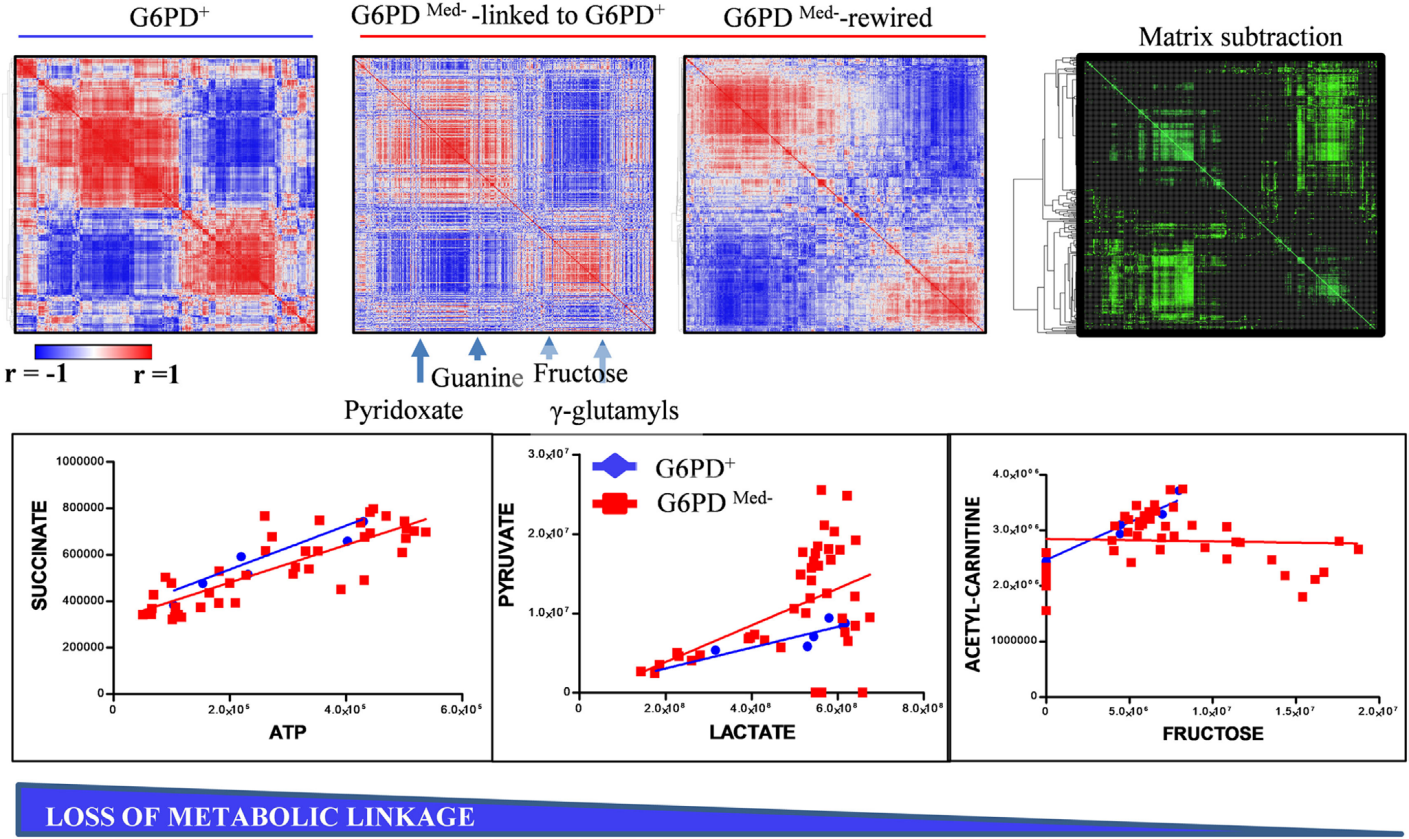
Metabolic linkage (35) analyses in glucose 6-phosphate dehydrogenase (G6PD)-deficient and sufficient red blood cells (RBCs). Metabolite levels were correlated to each other in both groups (independently of storage age), to identify variations (Δ|*r*| > 30%) in metabolite levels secondary to metabolic rewiring in G6PD-deficient donors when compared to G6PD sufficient controls. Phenotypic alterations that disrupted this fine tuning of the kinetics of specific metabolic pathways would be highlighted by a differential analysis of correlation of metabolites across conditions. Blue to red = −1 < *r* < +1. A highlight of correlations varying >30% between these two conditions is shown in green. For example, significant alteration of the linkage between lactate and pyruvate or acetyl carnitines and fructose, but not succinate and ATP, was observed in G6PD-deficient RBCs (red line and squares) vs. G6PD sufficient counterparts (blue lines and rhomboids).

Though correlations do not necessarily imply causation, in the field of metabolomics, a high degree of correlation is observed among the levels of metabolites from pathways that are linked by biochemical constraints of enzymatic reactions (35). The identification of such correlates under physiological conditions and the disruption of such correlations under pathological conditions (e.g., here G6PD deficiency, Figure 5) are indicative of metabolic rewiring. Here, for example, we identify alterations between the correlates of pyruvate/lactate ratios, suggestive of disrupted NADH/NAD+ homeostasis in G6PD-deficient subjects. This observation confirms and expands upon our previous report about increased levels (and potentially increased activity) of methemoglobin reductase in RBCs from G6DP^−^ donors (22). On the other hand, we also noted a disruption in the correlation between acetylcarnitine and fructose. Of note, RBC levels of carnitine and acetylcarnitine are, respectively, comparable and higher to the levels observed in plasma (36). Indeed, RBCs are equipped with a functional ATP-citrate lyase, as we (31, 37) and others (38) have shown with tracing experiments with ^13^C-glucose and other stable isotope tracers. Disruption of correlation between acetylcarnitine and fructose levels in G6DP^−^ donors is suggestive that, in normal RBCs, at least part of the acetylcarnitine pool is derived from fructose sugar and that this metabolic route is dysregulated in G6DP^−^ donors. Follow-up tracing experiments with ^13^C-fructose will be necessary to expand on this observation.

**Figure 6:**
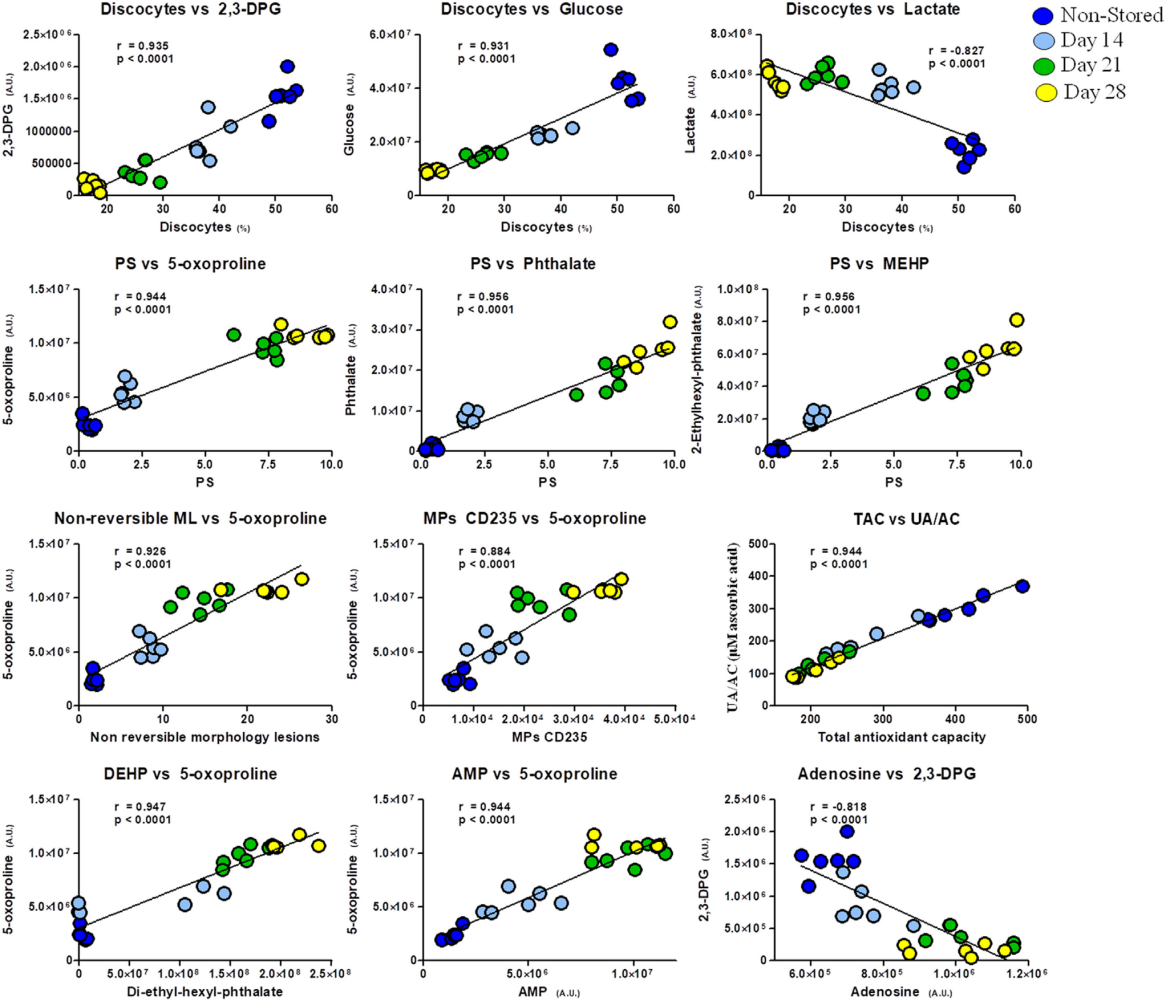
Correlation analysis of metabolites with physiological features and morphological outcomes in glucose 6-phosphate dehydrogenase-deficient red blood cells at different storage days (non-stored/fresh blood: dark blue; day 14: light blue; day 21: green; day 28: yellow dots).

In addition, apart from the intrametabolic correlates, several couples of correlations involving metabolites and physiological RBC/plasma characteristics can be identified in G6PD^−^ donors, physically linked to each other by exhibiting the same variation profile in fresh blood (non-stored, NS) and throughout storage in CPD/SAGM (Figure 6). Results are indicative of a correlation between the energy state of the stored RBC (as gleaned by 2,3-DPG, glucose, and lactate levels) and the preservation of a discocytic phenotype, while MPs release and phosphatidylserine exposure correlated with markers of impaired glutathione homeostasis and (maybe merely spuriously) with the total levels of phthalates measured at any given time point. Of note, correlations between AMP levels and gamma-glutamyl-cycle end-product 5-oxoproline (in oxoprolinase-deficient mature RBCs) is suggestive of an intertwinement between energy and redox metabolism, further confirming our recent reports on the role of oxidative stress in stored RBC energy impairment secondary to AMP deaminase activation (39).

### The Metabolic and Biopreservation Profiles of G6PD^−^ Packed RBCs Were Closely Related to the Biological Profile of the Donor: Intra-Parameter Relationships

**Figure 7:**
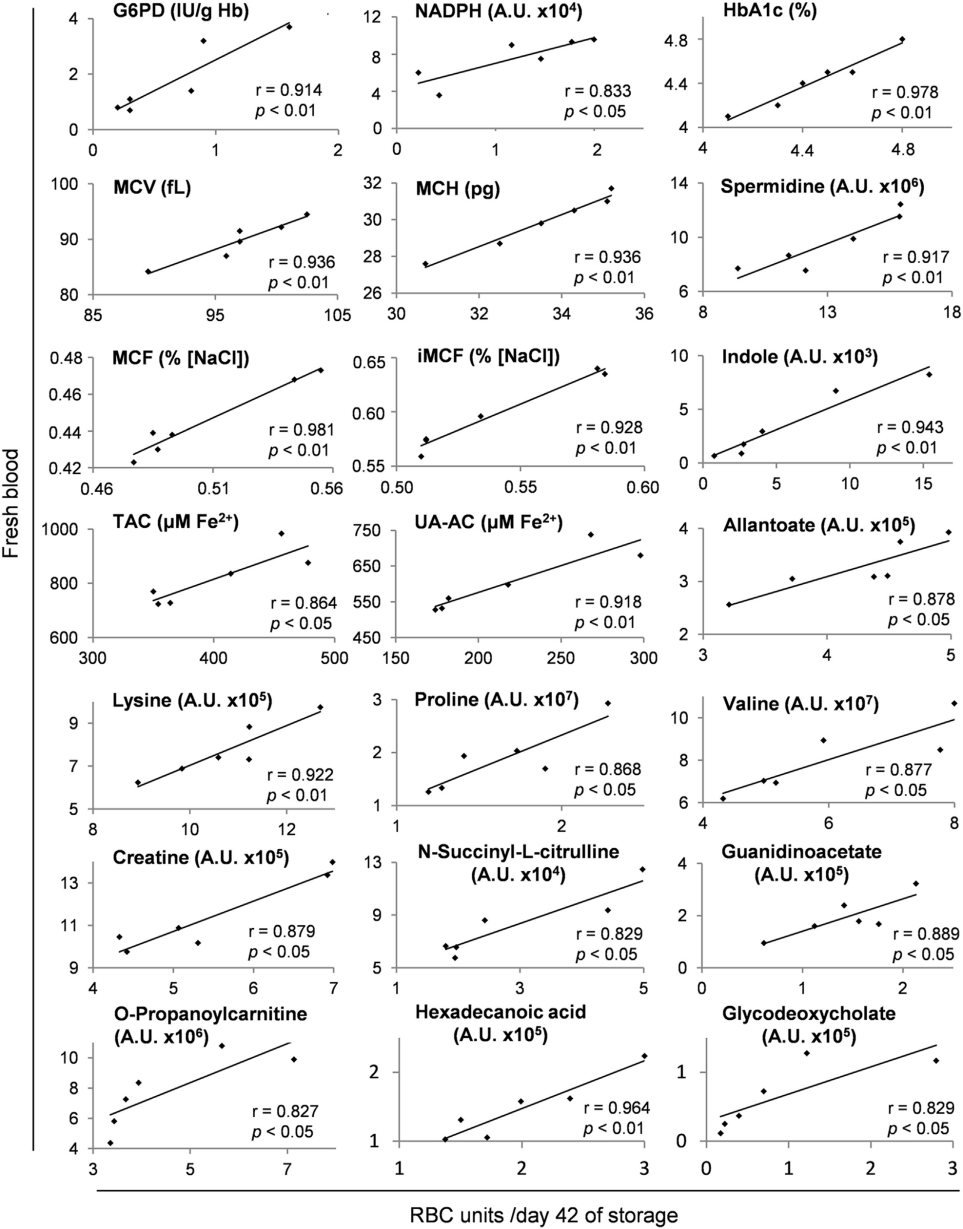
Representative scatter plots of Pearson’s correlations between hematological, physiological, and metabolism parameters in fresh donors’ blood and packed red blood cells (RBCs) of the same donors after 42 days of storage. Apart from day 42 shown here, these fresh blood variables had statistically significant correlations (see Materials and Methods) with those of stored RBC units at every time point of the storage period, as shown in Table S3 in Supplementary Material.

Several metabolites and physiological characteristics of G6PD^−^ packed RBCs fluctuate throughout the storage period proportionally to their own baseline levels *in vivo*, as shown in Table S3 in Supplementary Material (whole storage) and in the representative scatter plots of Figure 7 (end-of-storage). Among these, the decreasing over storage (22, 40) G6PD activity, reducing power (NADPH) and antioxidant capacity (23), along with the increasing glycated Hb (HbA1c) (41) and osmotic hemolysis levels (23) were included. Similar findings regarding the donor-dependent resistance of RBCs to osmotic lysis and the antioxidant capacity of the supernatant have been previously detected in packed RBCs from G6PD^+^ donors (14, 15, 42, 43), signifying a “donor-signature effect” on storage. Indeed, while their absolute values vary following variations in storage duration, mediums, and strategies, or in the genetic background (current study), their overall storage profile is steadily a function of donor levels. In other words, the blood banking affects them equally and by a “stable factor” of effect.

### Inter-Parameter Relationships and Networking

In addition to those “intra-parameter” relationships, more than 900 repeatable correlations observed between variables in fresh and stored RBCs, as shown in the G6PD^−^ specific *in vivo*-vs. -*ex vivo* biological network of Figure S2 in Supplementary Material. This complex structure is a correlation-based, strict assessment of distinct and multifarious experimental metrics, designed to minimize the unreliable interrelations, as each connection is statistically significant at 6 different time points of the storage period (a total of 36 replicates in the 6 G6PD^−^ units). Certain hub nodes represented specific storability variables that correlated to a large number of donor entities (and *vice versa*). The higher density of connections is observed at the upper right area of the network (I), where in-bag hemolysis and susceptibility to hemolysis (I-A) clustered with the 2,3-BPG (I-B) and dehydroascorbate (DHA, I-C) hub nodes. In a clockwise way, the areas of bile acids (II), extracellular K^+^ (III), G6PD activity (IV), redox (including ROS, urate, and antioxidant capacity, V), hematological (MCV, MCH, HbA_1c_, VI), and fatty/bile acids (VII) can be observed (Figure S2 in Supplementary Material). To focus on entities intrinsically related to the G6PD deficiency, the progress of storage lesion (e.g., redox status) and the quality of RBC concentrates (e.g., hemolysis-related variables) fragmentary analyses of sub-networks were subsequently performed.

### G6PD and Metabolic Networking Revealed that RBC Storage Biology Is Physically Related to Donor Biology, though at a Broader Level of Interwoven Underlying Pathways

**Figure 8:**
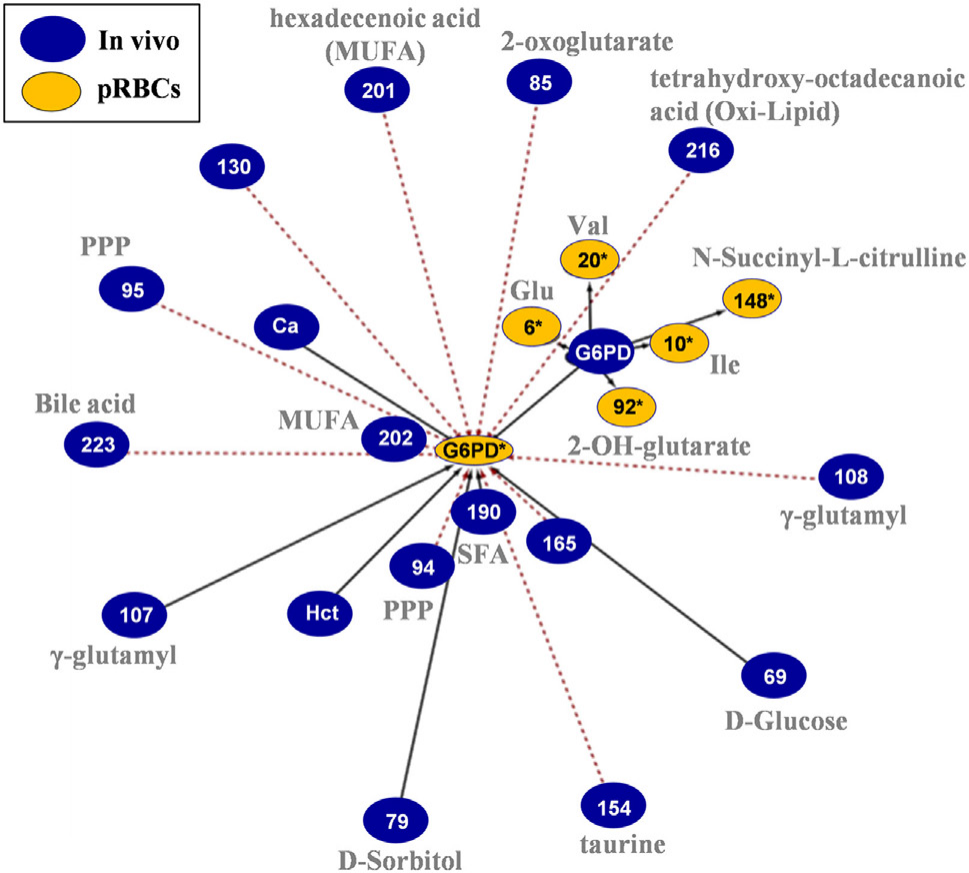
*In vivo* (fresh blood) vs. *ex vivo* [packed red blood cells (RBCs)] network analysis of glucose 6-phosphate dehydrogenase (G6PD) activity in G6PD^−^ donors. The G6PD activity sub-network consists of 23 statistically significant and repeatable at any storage duration correlations (see Materials and Methods). Same as all networks currently reported, only the correlations of Day 42 with fresh blood measurements are shown; however, those pairs of statistically significant connections (with slightly different *r*) applied to all possible storage durations (Days 7, 14, 21, 28, 35, and 42). The length of each line is inversely proportional to the *r* value of the correlation (the shorter the edge, the higher *r* value). Continuous black lines: positive correlations; dashed red lines: negative correlations.

In the G6PD activity network (Figure 8), the baseline levels had positive correlations with the in-bag levels of amino acids and 2-OH-glutarate. On the other side, in-bag G6PD activity had inverse correlations with metabolites of the PPP cycle, monounsaturated fatty acids, bile acids, and oxidized lipids in fresh blood.

Regarding the main metabolic pathways, the end-products of glycolysis (Figure S3 in Supplementary Material) *in vivo* had negative correlations with the levels of 2,3-BPG but opposite correlations with numerous hemolysis/fragility parameters of the RBC concentrates and supernatant K^+^. In the same way, in the PPP and one-carbon metabolism pathways (Figure S4 in Supplementary Material), sedoheptulose-1-phosphate, and folate levels in fresh RBCs had positive correlations with in-bag levels of 2,3-BPG and redox variables (DHA) but inverse correlations with hemolysis-related metrics. An interesting link between *in vivo* levels of protein carbonylation and osmotic fragility with the reducing power of the packed RBCs was also noticed. In reverse, *in vivo* levels of glucono-1,5-lactone-6P had strong correlations with those of the UA-dependent antioxidant capacity of the supernatant and of the G6PD activity. In the glutathione cycle, transaminases, and malate-aspartate shuttle, the majority of connections concerned *in vivo* GSH/GSSG content, fumarate, and malate toward stored RBCs’ amino acid and fatty acids metabolism, DHA, malate, 2,3-BPG, hemolysis, and extracellular K^+^.

Consistent with the involvement of G6PD (through the production of NADPH) in the reductive biosynthesis of fatty acids and cholesterol (44), two-way ANOVA processing of the metabolomics analyses revealed clear differences in lipid content, biosynthesis, and metabolism between G6PD^−^ and control RBCs both *in vivo* (e.g., palmitate, oleate) and during storage (e.g., glycerol phosphate, eiosatrienoate, arachidonate) (Figure 3), in a way likely affecting recoveries, as shown in multiple mouse strains (16, 25). In those studies, lipid metabolism, degradation and oxidation emerged as the strongest correlates with poor 24-h recoveries. Moreover, in both animal and human transfusion contexts, bioactive lipid components of the transfusate are considered clinically relevant for the transfused recipients (45). Subsequent network analysis (Figure S5 in Supplementary Material) further verified the central role of lipids in defining the demanding membrane properties and thus, the quality of stored G6PD^−^ RBCs (5, 25). The levels of linoleate, thromboxane B2, and leukotriene C4 had inverse correlations with those of 2,3-BPG and DHA in the RBC unit, while those of eicosapentaenoic/eicosatetraenoic and hexadecanoic acids at donation correlated well (negatively) with the fragility of stored RBCs and the concentration of extracellular potassium in the supernatant.

The interesting interplay among the G6PD-affected metabolic pathways (e.g., NAD+/glucose-6-phosphate, GSH/2,3-BPG) before and during refrigerated storage, suggests that RBC storage biology reflects a part of donor biology, in a way hardly revealed by individual factor metrics. Lactate levels in the G6PD^−^ donors, for example, were not proportional to lactate levels in stored RBC units, but rather with those of the pathway-interconnected 2,3-BPG.

### The Quality of the Biopreservation of the G6PD^−^ RBC Unit Is Linked by Biochemical and Cellular Pathways with the *In Vivo* State

**Figure 9:**
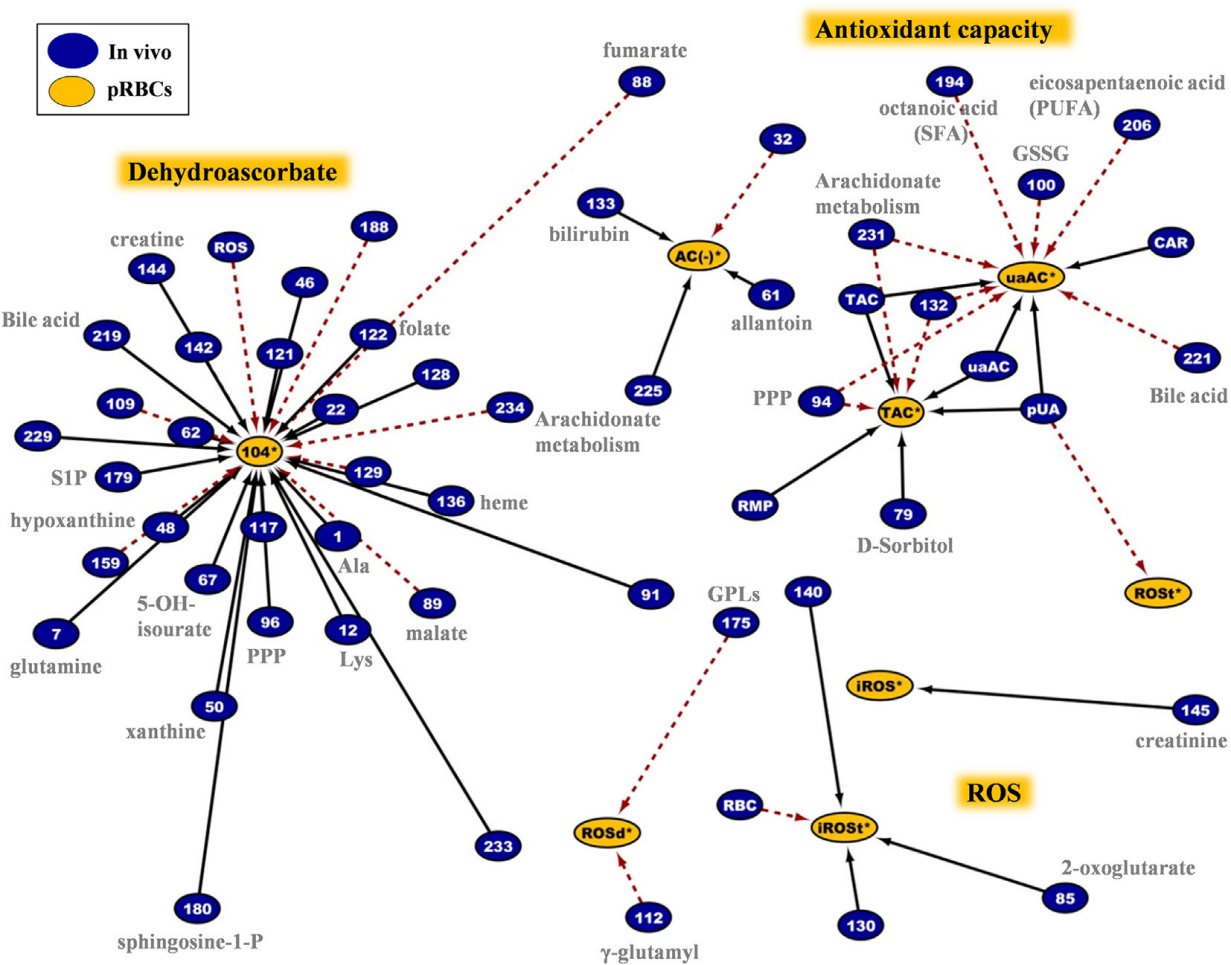
Network analysis of donors’ variables at time of donation that had significant correlations with the redox status of the packed red blood cells (RBCs) regardless of the storage duration. The sub-network consists of 62 connections involving intracellular reactive oxygen species (ROS) accumulation, antioxidant capacity of the supernatant, DHA, and other variables. In-bag DHA and ROS levels at post-storage-mimicking conditions represented the most important hub nods. Plasma uric acid and its attributed antioxidant capacity revealed their “intra-parameter” dynamics (see also Figure 7). Continuous black lines: positive correlations; dashed red lines: negative correlations.

The network analyses made clear that the redox and hemolysis-related parameters of the G6PD^−^ RBC unit are among those most correlated with the *in vivo* state. These variables characterize not only the storability of G6PD^−^ RBCs but also a part of their post-transfusion performance. The sub-interactome shown in Figure 9 contains the sum of the *in vivo* physiological and omics variables having correlations with the in-bag levels of oxidant/antioxidant variables. According to this map, donor levels of RBC ROS had no correlation with those of stored RBCs, but they did have a strong inverse correlation with DHA levels, throughout the storage period. Reversely, susceptibility of stored RBCs to thermal- or oxidant-induced ROS generation had strong correlations with the levels of serum UA in fresh blood. In addition, the antioxidant capacity of the supernatant had positive correlations with the *in vivo* levels of UA/allantoin, PPP components, GSH, arachidonate, and fatty acid metabolism. It seemed that high levels of GSSG in fresh blood predispose the RBC unit to low levels of UA-dependent antioxidant activity throughout the storage period. Storage DHA was the most important hub nod in the redox network. It showed positive correlations with amino acids, nucleotides, GPLs such as sphingosine-1-phosphate, prostaglandin D3, leukotriene A4, and folate, but opposite correlations with the levels of intracellular ROS, fumarate, malate, and leukotriene C4 at donation.

**Figure 10:**
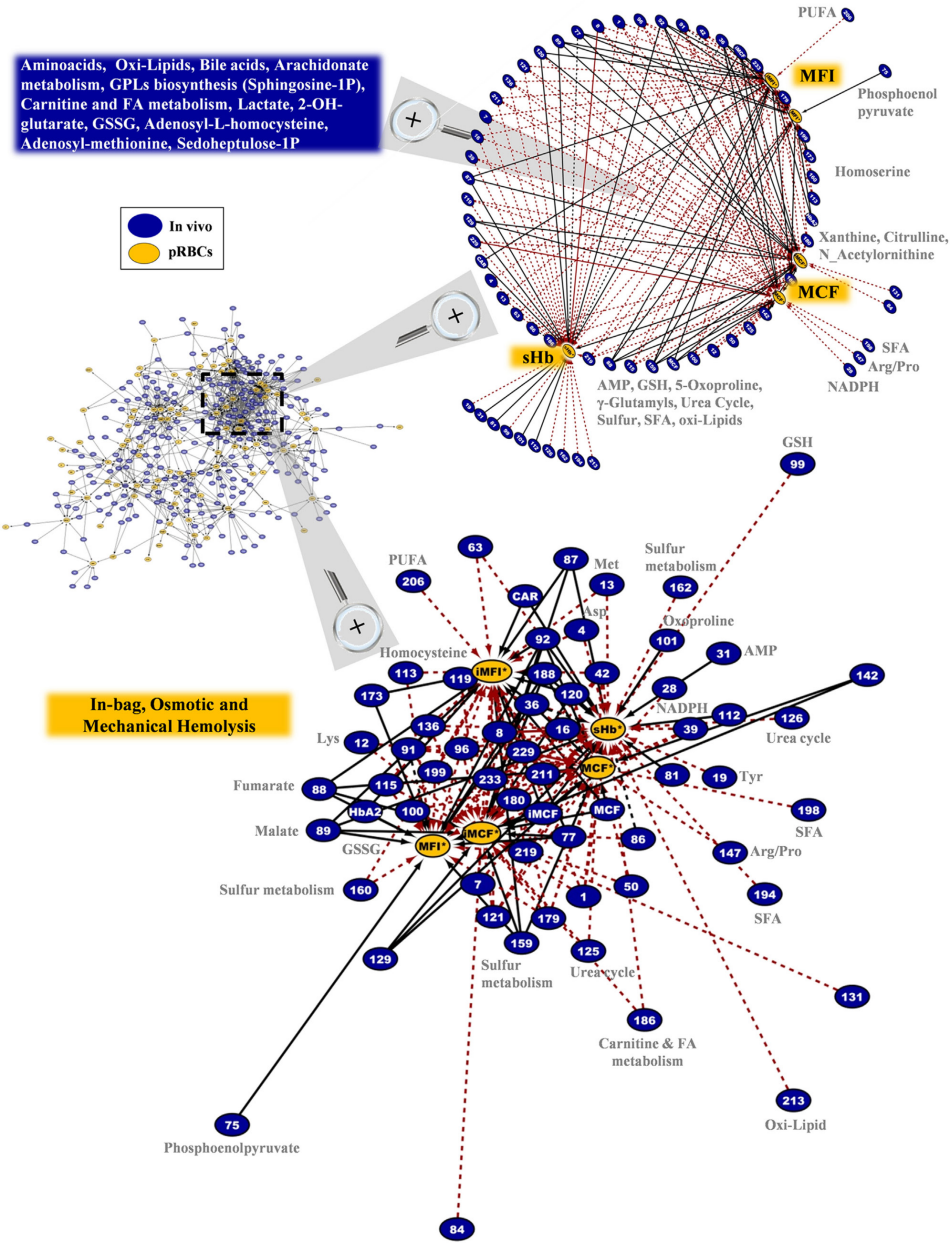
In-bag hemolysis (sHb) and the resistance of stored G6PD^−^ red blood cells (RBCs) to osmotic or mechanical lysis had several correlations with the *in vivo* state. Magnification of the most dense network cluster (dashed frame) that contains the hemolysis-related nodes of the packed RBCs. *Upper panel*: circle layout of the donor variables reveals that only few of them had correlations with individual hemolysis metrics in packed RBCs (right side of the map), as the majority are connected with at least two of them (blue box in the left side). In this layout, the length of the connections is unrelated to the correlation coefficient *r* value; however, the positive/negative correlations follow the color code shown in Figure 8 (black-solid/red-dashed, respectively). *Down panel*: the interactome connecting hemolysis-related nodes by 173 repeatable correlations captures a part of the polyparametricity of the “hemolysis” phenotype and the “intra-parameter” dynamics of RBC osmotic fragility both *in situ* (MCF) and following incubation for 24 h at 37°C (iMCF). Continuous black lines: positive correlations; dashed red lines: negative correlations.

Regarding the hemolytic variables of the RBC units in G6PD deficiency, an impressive polyparametric sub-network of 173 connections links them to the *in vivo* state (Figure 10). The network refers to in-bag levels of hemolysis and to the osmotic/mechanical fragility of stored RBCs *in situ* or in post-storage-simulating conditions. A few donor variables had individual correlations with in-bag hemolysis (e.g., AMP, 5-oxoproline, GSH), or osmotic hemolysis (e.g., NADPH, xanthine, citrulline), or mechanical hemolysis (e.g., homocysteine); however, the vast majority of them correlated (positively or negatively) with most of, or all, the hemolysis-related variables of packed RBCs (*r*-independent circular network in the upper panel of Figure 10). Thus, increased levels of amino acids, lipids, and lipid metabolism factors (including sphingosine-1-phosphate and prostaglandin D3), serine biosynthesis metabolites, NADPH, and GSH, among others *in vivo* predispose RBCs to better storability profiles (*r*-driven sub-network in the down panel of Figure 10). A clear opposite trend was seen for the protein carbonylation, osmotic fragility, AMP (see also purines involving network in Figure S6 in Supplementary Material), lactate, malate, 2-OH-glutarate (that is a marker of hypoxia), fumarate, and oxoproline. In the hemolysis network, certain donor metabolites are components of the one-carbon and sulfur metabolism (see also Figure S7 in Supplementary Material) that were found at lower (e.g., methionine) or higher (e.g., homocysteine) levels in the G6PD^−^ donors compared to controls (Figure 2). It is worth noting that while G6PD activity *in vivo* had no correlation itself with in-bag hemolysis or cellular fragilities, several G6PD-related metabolites (GSH, NADPH, etc.) had strong correlations with at least one hemolysis-related node, verifying the significantly higher analytical power of metabolomics compared to one-molecule-targeted biochemical approaches for probing cellular physiology (46).

**Figure 11:**
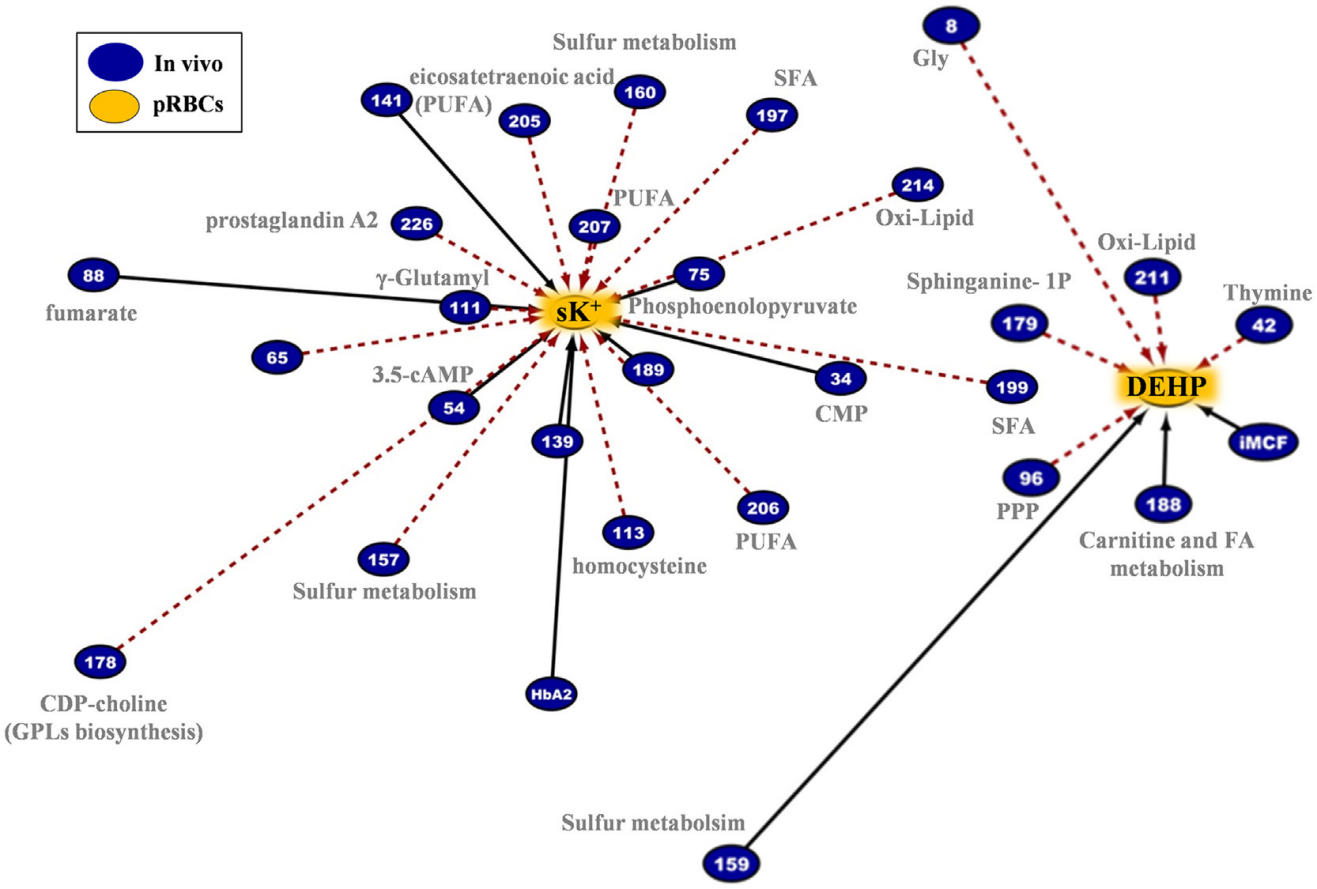
Extracellular potassium and bis(2-ethylhexyl) phthalate (DEHP) connections in pRBCs donated by G6PD^−^ donors. Lipids and lipid metabolism constitute significant contributors in both networks. Continuous black lines: positive correlations; dashed red lines: negative correlations.

The G6PD^−^ RBCs are more susceptible than the G6PD^+^ RBCs to K^+^ leak (22), which potentially increases the risk of hyperkalemia-induced arrhythmia in susceptible recipients (47). End-of-storage extracellular potassium had numerous negative correlations with donor metabolites in fresh RBCs, most of which in the categories of fatty acids and lipid metabolism or metabolites potentially involved in lipid and protein oxidation (e.g., the highly expressed L-homocysteine) (Figure 11, *left panel*). As in the case of in-bag hemolysis and mechanical fragility, high *in vivo* levels of fumarate, phosphoenolopyruvate, and hydroxybutyrate correlated with high in-bag potassium concentration. The progressive degradation of RBC membrane with storage apparently leads to increasing levels of extracellular Hb (as both free Hb and extracellular vesicles) and potassium. Of note, donor levels of AMP were negatively associated with both variables. Finally, the phthalate-specific network support the above mentioned hypothesis that the distinct lipid composition and mechanical properties of the membrane in G6PD deficiency may drive the differential incorporation of DEHP in the bilayer of stored RBCs (Figure 11, *right panel*).

### Hemolysis Is a Multivariate “Phenotype” of the RBC Storage Lesion, Functionally Connected to Donor Biology by More than One Tethers

Strikingly enough, donor levels of free Hb and RBC mechanical fragility did not correlate with any of the hemolysis-related variables of the RBC concentrates (Figure 10). According to a number of metabolomic studies, genetic factors contribute substantially to the degree of storage hemolysis (5), while several donor-specific “metabotypes” have been described in stored RBCs (48). Hemolysis, however, is a multivariate phenotype of the stored RBCs and our study revealed only a fraction of the multitude and the complexity of the donor factors that likely affect it. Without doubt, additional omics analyses (proteomics, lipidomics, etc.) would further elucidate the phenomenon. The biological complexity is too high and the *in vitro* system substantially different compared to the *in vivo* state where the homeostatic mechanisms of healthy RBCs were evolved to meet the cell integrity needs. In-bag hemolysis and post-transfusion recovery represent the overall effect of storage- and recipient-related stresses on distinct physiological characteristics of RBCs that might be donor related. For instance, the osmotic fragility of CPDA-stored RBCs has a correlation with the levels of in-bag hemolysis, but, in quantity terms, fragility can reveal no more than 11% of its variation (42). Moreover, stored RBCs from G6PD^+^ donors that repeatedly exhibit high in-bag hemolysis at outdate are also characterized by reduced ability to resist osmotic stress compared to those exhibiting normal hemolysis (49). In a similar way, in-bag hemolysis in G6PD^−^ units had a positive correlation with the baseline levels of osmotic hemolysis at body temperature (Figure 10).

### Interplay of Redox, Energy, and Hemolysis-Related Factors before and during Storage

The hemolytic phenotype is likely based on changes in redox and energy metabolism that affect the deformability and stability of RBCs under physiological or pathologically levels of stress (50). The redox activity of RBCs, which contain a strongly oxidizing cytoplasm, governs their lifetime in circulation (51, 52), and thus, changes in the antioxidant activity during storage (53) may have a substantial effect on pre- and post-storage viability. Quantitative proteomics analyses have identified a number of proteins in the supernatant of RBC units showing linear correlations with the absolute levels of extracellular Hb (54), while the donor-related susceptibility of stored RBCs to hemolysis was associated with modifications in RBC membrane proteins involved in oxidative response pathways and decreased storage levels of 2,3-BPG (49). The present study in G6PD^−^ RBCs, which are more susceptible to metabolic changes and protein oxidation compared to normal cells (22, 24), further revealed the strong interplay of in-bag hemolysis and donor biology, since, for instance, RBC protein oxidation at baseline had positive correlations with both in-bag and mechanical hemolysis at body temperature (Figure 10).

Moreover, the levels of a relatively homogeneous panel of metabolites in fresh blood (including amino acids, carboxylic acids, GPLs, fatty acids, and purine metabolism components) strongly predispose RBCs to either good or poor storage. Previous studies in stored RBCs have identified some of them (e.g., amino acids) as having similar trend of correlations (negative) with the same-day hemolysis levels (5). In our study, sphingosine-1-phosphate showed positive correlation with the quality of the RBC unit. This bioactive signaling lysophospholipid, is functionally related to RBC (55, 56) and transfusion biology (57) as having critical roles in blood homeostasis and vascular permeability (58). The interconnections of redox, energy, and hemolysis parameters before and during storage (e.g., lactate/hemolysis, GSH/hemolysis, NADPH/osmotic fragility, ROS/2,3-BPG in fresh/stored samples, respectively), further highlight the usefulness of omics/bioinformatics analyses in revealing the complexity of the RBC storage lesion as a function of inter-donor variability and its underlying mechanisms.

### Some Pieces of the Interactomes Might Be Related with the Post-Transfusion Performance of G6PD-Deficient RBCs

According to previous reports, the G6PD^−^ RBCs exhibited normal levels of ROS and in-bag hemolysis; however, exposure to post-transfusion mimicking conditions, including recipient plasma, body temperature (37°C), and oxidants (24), promote hemolysis and ROS accumulation compared to control RBCs (22). The deformability of stored RBCs may determine their quality and post-transfusion performance (59), while *in vitro* testing of RBC responses in recipient-mimicking contexts would reveal clinically relevant sublethal injuries of transfused RBCs (2, 60, 61). In this context, it was interesting that the redox network of Figure 9 includes several responses of stored RBCs to oxidants (tBHP, diamide) that may indeed interfere with the function of stored RBCs in recipients characterized by (medication- or infection-induced) redox disequilibrium. Moreover, variation in the RBC osmotic fragility throughout the storage period was found proportional to its baseline levels in G6PD^−^ donors (Figure 10). In a similar way, numerous biochemical components *in vivo* (including S-adenosyl methionine that provides cysteine to support GSH synthesis) had significant correlations with the fragilities of stored G6PD^−^ RBCs after 24 h staying at body temperature (iMCF, iMFI). Of note, alpha-tocopherol levels in fresh mouse RBCs were reported to correlate with recovery in animal studies (25) and its levels in G6PD^−^ donors correlated not only with in-bag hemolysis but also with the pro-hemolytic features of the stored RBCs. Our findings may be functionally linked to the performance of G6PD^−^ RBCs, which has been questioned at both laboratory (22) and clinical levels (21). To support, *in vivo* levels of aspartate, glutamine and thymine were indeed reported to correlate with recovery in mice (25). Despite the fact that these correlations should be confirmed by *in vivo* studies in human, they provide an insight into the pathophysiology of post-transfusion complications in therapies involving G6PD^−^ donors.

## Conclusion

This study showed for the first time that the metabolic phenotypes of G6PD-deficient donors recapitulate the basic storage lesion profile observed in G6PD sufficient donors, which is characterized by loss of metabolic linkage and rewiring, in spite of certain differences observed in one-carbon metabolism, glutathione/urate homeostasis, and fatty acid pathways. Moreover, it revealed that donor variability issues affect the storage quality even in the narrow context of this small donor subgroup characterized by an enzymatic genetic defect. We reported an interesting and informative interplay between redox, energy, and hemolysis parameters before and during storage, namely, between factors which differ among G6PD^−^ donors at the time blood was harvested, and the storability of donated RBCs, a part of which may be related to their performance in the transfused patient. Our data provide mechanistic insight into the biology of RBC storage in G6PD deficiency and could guide future studies focusing on donor biology-related factors involved in the regulation of storage-induced hemolysis that is a multivariate phenotype of the RBC storage lesion. Development of reliable physiological and metabolic biomarkers of storage quality and post-transfusion performance (e.g., post-transfusion recovery, iron metabolism) in fresh or stored blood from G6PD sufficient or deficient donors would allow donor screening and thus improved management of both donor and RBC inventory at time of collection or prior to release of the RBC concentrates from the Blood Bank.

## Ethics Statement

The study was approved by the Ethics Committee of the Department of Biology, School of Science, NKUA. Investigations were carried out upon signing of written consent, in accordance with the principles of the Declaration of Helsinki.

## Author Contributions

Each author has contributed to the submitted work as follows: AK, AD, and MA designed the study. JR, TN, and AD performed the UHPLC-MS analyses. VT, AK, and MA prepared the RBC units and performed the hematological and physiological analyses. AV performed the biological networks. VT, AD and MA analyzed the results, prepared the figures, and wrote the first draft of the manuscript. IP critically commented on the interpretation of data and drafting of the manuscript, and all the authors contributed to the final version.

## Conflict of Interest Statement

The authors declare that the research was conducted in the absence of any commercial or financial relationships that could be construed as a potential conflict of interest. Though unrelated to the contents of this study, the authors disclose that AD and TN are members of Omix Technologies, Inc. The reviewer GP declared a past collaboration with one of the authors AD to the handling Editor.

## Supplementary Material

Table S1 Metabolomic analysis of G6PD sufficient (control) and G6PD deficient (G6PD def.) leukoreduced red blood cell concentrates throughout the storage period in CPD-SAGM.
