# Supplementary material for: Metabolic Linkage and Correlations to Storage Capacity in Erythrocytes from Glucose 6-Phosphate Dehydrogenase-Deficient Donors
Source: Front Med (Lausanne). 2018 Jan 11;4:248. doi: 10.3389/fmed.2017.00248 (PMC5768619; doi:10.3389/fmed.2017.00248)
Supplement: Supplementary file 2 [file Table_2.docx]

Supplementary Material

**Metabolic linkage and correlations to storage capacity in erythrocytes from glucose 6-phosphate dehydrogenase deficient donors**

Julie A. Reisz^1†^, Vassilis L. Tzounakas^2†^, Travis Nemkov^1^, Artemis I. Voulgaridou^3^, Issidora S. Papassideri^2^, Anastasios G. Kriebardis^4*^, Angelo D’Alessandro^1*^, Marianna H. Antonelou^2^

^1^University of Colorado, School of Medicine,, Department of Biochemistry and Molecular Genetics, Anschutz Medical Campus, Aurora, CO, USA

^2^ National and Kapodistrian University of Athens, School of Science, Department of Biology, Athens, Greece

^3^“Apostle Paul” Educational Institution, Thessaloniki, Greece

^4^Technological and Educational Institute of Athens, Faculty of Health and Caring Professions, Department of Medical Laboratories, Athens, Greece

*** Correspondence:**Anastasios Kriebardis
[akrieb@biol.uoa.gr](mailto:akrieb@biol.uoa.gr)

Angelo D’Alessandro

[^ANGELO.DALESSANDRO@UCDENVER.EDU^](mailto:ANGELO.DALESSANDRO@UCDENVER.EDU)

^†^equal first authors

| **SUPPL. TABLE 2** Abbreviations and annotations used in the biological networks | |
| --- | --- |
| **Parameter** | **Abbreviation** |
| **Hematological and physiological** |  |
| Uric acid-independent antioxidant capacity | AC(-) |
| Alanine transaminase | ALT |
| Aspartate transaminase | AST |
| Atheromatic index | ATH |
| Intracellular Ca^2+^ | Ca |
| Protein carbonylation (RBC membrane) | CAR |
| Total cholesterol | Chol |
| Ferritin | Fer |
| G6PD activity | G6PD |
| Total Hb (g/dL) | Hb |
| Glycated Hb | HbA1c |
| Hemoglobin A2 | HbA2 |
| Hemoglobin F (fetal) | HbF |
| Hematocrit | Hct |
| Incubated mean cell fragility RBCs | iMCF |
| Low-density lipoprotein | LDL |
| Mean corpuscular fragility | MCF |
| Mean corpuscular Hb | MCH |
| Mean corpuscular Hb concentration | MCHC |
| Mean corpuscular volume | MCV |
| Mechanical fragility index | MFI |
| Microparticles concentration (by FC) | MP |
| MP-associated procoagulant activity | MPPA |
| Non-reversible RBC-shape modifications | NR |
| Plasma free Hb (hemolysis) | pHb |
| Serum UA | pUA |
| Reversible RBC-shape modifications | R |
| RBC count | RBC |
| Reticulocyte % | RET |
| RBC-derived MPs | RMP |
| Reactive oxygen species | ROS |
| Total antioxidant capacity | TAC |
| Total bilirubin | T-BIL |
| UA-dependent Antioxidant Capacity | uaAC |
| **Aminoacids** |  |
| alanine | 1 |
| arginine | 2 |
| asparagine | 3 |
| aspartate | 4 |
| cysteine | 5 |
| glutamate | 6 |
| glutamine | 7 |
| glycine | 8 |
| histidine | 9 |
| isoleucine | 10 |
| leucine | 11 |
| lysine | 12 |
| methionine | 13 |
| phenylalanine | 14 |
| proline | 15 |
| serine | 16 |
| threonine | 17 |
| tryptophan | 18 |
| tyrosine | 19 |
| valine | 20 |
| cystine | 21 |
| kynurenine | 22 |
| **Phosphates** |  |
| Orthophosphate | 23 |
| Diphosphate | 24 |
| Triphosphate | 25 |
| **Nucleotides** |  |
| NAD+ | 26 |
| NADH | 27 |
| NADPH | 28 |
| NADP+ | 29 |
| ADP | 30 |
| AMP | 31 |
| UDP-glucose | 32 |
| GDP | 33 |
| CMP | 34 |
| CTP | 35 |
| UTP | 36 |
| UMP | 37 |
| IMP | 38 |
| Uracil | 39 |
| Adenine | 40 |
| Nicotinamide | 41 |
| Thymine | 42 |
| Adenosine | 43 |
| Thymidine | 44 |
| Adenylyl sulfate | 45 |
| Guanine | 46 |
| Pyridoxal | 47 |
| Hypoxanthine | 48 |
| Inosine | 49 |
| Xanthine | 50 |
| Guanosine | 51 |
| Cytidine | 52 |
| Allantoate | 53 |
| 3--5--Cyclic AMP | 54 |
| cyclic GMP | 55 |
| Pyridoxamine phosphate | 56 |
| 4-Pyridoxate | 57 |
| 5-6-Dihydrothymine | 58 |
| 3--5--Cyclic IMP | 59 |
| Nicotinate ribonucleotide | 60 |
| (S)(+)-Allantoin | 61 |
| 2--3--Cyclic CMP | 62 |
| 3-Ureidopropionate | 63 |
| 5-Phosphoribosylamine | 64 |
| 5--Phosphoribosyl-N-formylglycinamide | 65 |
| 3-Ureidoisobutyrate | 66 |
| 5-Hydroxyisourate | 67 |
| Purine | 68 |
| **Glycolysis** |  |
| D-Glucose | 69 |
| D-Glucose 6-phosphate | 70 |
| D-Fructose 1-6-bisphosphate | 71 |
| D-Glyceraldehyde 3-phosphate | 72 |
| 2-3-Bisphosphoglycerate | 73 |
| 2/3-Phospho-D-glycerate | 74 |
| Phosphoenolpyruvate | 75 |
| Pyruvate | 76 |
| Lactate | 77 |
| Mannitol | 78 |
| D-Sorbitol | 79 |
| D-Ribose | 80 |
| D-Rhamnose | 81 |
| D-Fructose | 82 |
| **TCA cycle** |  |
| cis-Aconitate | 83 |
| Citrate | 84 |
| 2-Oxoglutarate | 85 |
| 2-Oxoglutaramate | 86 |
| Succinate | 87 |
| Fumarate | 88 |
| Malate | 89 |
| Oxaloacetate | 90 |
| **Alternative Carboxylic acids** |  |
| 5-Aminolevulinate | 91 |
| 2-Hydroxyglutarate/Citramalate | 92 |
| **Pentose phosphate pathway** |  |
| 6-Phospho-D-gluconate | 93 |
| D-Glucono-1-5-lactone 6-phosphate | 94 |
| D-Erythrose 4-phosphate | 95 |
| Sedoheptulose 1-phosphate | 96 |
| alpha-D-Ribose 1-phosphate | 97 |
| 5-Phospho-alpha-D-ribose 1-diphosphate | 98 |
| **GSH homeostasis** |  |
| Glutathione | 99 |
| Glutathione disulfide | 100 |
| 5-Oxoproline | 101 |
| S-Glutathionyl-L-cysteine | 102 |
| Ascorbate | 103 |
| Dehydroascorbate | 104 |
| **Gamma-glutamyls** |  |
| gamma-L-Glutamyl-L-cysteine | 105 |
| gamma-Glutamyl-Se-methylselenocysteine | 106 |
| gamma-L-Glutamyl-D-alanine | 107 |
| gamma-Glutamyl-gamma-aminobutyrate | 108 |
| (5-L-Glutamyl)-peptide | 109 |
| (5-L-Glutamyl)-L-glutamine | 110 |
| 5-L-Glutamyl-taurine | 111 |
| gamma-L-Glutamylputrescine | 112 |
| **Serine biosynthesis and one-carbon metabolism** |  |
| L-Homocysteine | 113 |
| Cystathionine | 114 |
| Homoserine | 115 |
| N-N-Dimethylglycine | 116 |
| D-O-Phosphoserine | 117 |
| 3-Phosphonooxypyruvate | 118 |
| S-Adenosyl-L-homocysteine | 119 |
| S-Adenosyl-L-methionine | 120 |
| Sarcosine | 121 |
| Folate | 122 |
| 5-10-Methenyltetrahydrofolate | 123 |
| **Urea cycle** |  |
| Ornithine | 124 |
| L-Citrulline | 125 |
| N-(L-Arginino)succinate | 126 |
| Urate | 127 |
| **Polyamines** |  |
| Cadaverine | 128 |
| Putrescine | 129 |
| Spermidine | 130 |
| Spermine | 131 |
| **Heme synthesis/turn-over** |  |
| Biliverdin | 132 |
| Bilirubin | 133 |
| Heme | 134 |
| Bilirubin-glucuronoside | 135 |
| Heme A | 136 |
| Heme C | 137 |
| Heme O | 138 |
| **Aminosugars** |  |
| N-Acetylneuraminate | 139 |
| alpha-D-Glucosamine 1-phosphate | 140 |
| L-Arabinose | 141 |
| 1-4-beta-D-Xylan | 142 |
| **Arginine and proline metabolism** |  |
| Phosphocreatine | 143 |
| Creatine | 144 |
| Creatinine | 145 |
| 4-Acetamidobutanoate | 146 |
| N-Acetylornithine | 147 |
| N-Succinyl-L-citrulline | 148 |
| Guanidinoacetate | 149 |
| trans-4-Hydroxy-L-proline | 150 |
| **Panthothenate metabolism** |  |
| Pantetheine | 151 |
| Pantetheine 4--phosphate | 152 |
| Pantothenate | 153 |
| **Sulfur metabolism** |  |
| Taurine | 154 |
| 3-Sulfino-L-alanine | 155 |
| Mercaptopyruvate | 156 |
| 2S-5S-Methionine sulfoximine | 157 |
| L-Selenomethionine | 158 |
| 3-Sulfocatechol | 159 |
| L-Cysteate | 160 |
| (R)-S-Lactoylglutathione | 161 |
| L-Methionine S-oxide | 162 |
| Cys-Gly | 163 |
| **Indole and Tryptophan** |  |
| Indole | 164 |
| 6-Hydroxykynurenic acid | 165 |
| **Inositol** |  |
| Ectoine | 172 |
| 3D-(3-5/4)-Trihydroxycyclohexane-1-2-dione | 173 |
| **Glycerophospholipid biosynthesis** |  |
| sn-Glycerol 3-phosphate | 174 |
| Ethanolamine phosphate | 175 |
| sn-glycero-3-Phosphoethanolamine | 176 |
| Diacylglycerylhomoserine | 177 |
| CDP-choline | 178 |
| Sphinganine 1-phosphate | 179 |
| Sphingosine 1-phosphate | 180 |
| **Carnitine and fatty acid metabolsim** |  |
| L-Carnitine | 181 |
| O-Acylcarnitine | 182 |
| O-Acetylcarnitine | 183 |
| O-Propanoylcarnitine | 184 |
| O-Butanoylcarnitine | 185 |
| L-Octanoylcarnitine | 186 |
| O-Decanoyl-L-carnitine | 187 |
| Acetoacetate | 188 |
| Hydroxybutyrate | 189 |
| **Saturated Fatty acids** |  |
| Butanoic acid | 190 |
| Pentanoate (valerate) | 191 |
| Hexanoic acid (caproate) | 192 |
| Heptanoic acid | 193 |
| Octanoic acid (caprylate) | 194 |
| Decanoic acid (caprate) | 195 |
| Dodecanoic acid | 196 |
| Tetradecanoic acid | 197 |
| Hexadecanoic acid | 198 |
| Octadecanoic acid | 199 |
| **Monounsaturated Fatty Acids** |  |
| Tetradecenoic acid | 200 |
| Hexadecenoic acid | 201 |
| Octadecenoic acid | 202 |
| **Poly-unsaturated Fatty Acids** |  |
| Linoleate | 203 |
| Octadecatrienoic acid | 204 |
| Icosatetraenoic acid | 205 |
| Icosapentaenoic acid | 206 |
| Docosahexaenoic acid | 207 |
| Dodecanedioic acid | 208 |
| **Essential fatty acids** |  |
| (8Z-11Z-14Z)-Icosatrienoic acid | 209 |
| (7Z-10Z-13Z-16Z-19Z)-Docosa-7-10-13-16-19-pentaenoic acid | 210 |
| **oxi-Lipids** |  |
| alhpa-tocopheronolactone | 211 |
| dihydroxy-octadecanoic acid | 212 |
| hydroxy-dodecanedioic acid | 213 |
| dihydroxy-hexadecanoic acid | 214 |
| trihydroxy-octadecenoic acid | 215 |
| tetrahydroxy-octadecanoic acid | 216 |
| **Bile acids** |  |
| 3alpha-7alpha-12alpha-Trihydroxy-5beta-cholanate | 217 |
| Glycocholate | 218 |
| Taurocholate | 219 |
| Taurochenodeoxycholate | 220 |
| 3alpha-12alpha-Dihydroxy-5beta-cholanate | 221 |
| Glycodeoxycholate | 222 |
| Glycochenodeoxycholate | 223 |
| Sulfoglycolithocholate | 224 |
| **Arachidonate metabolism** |  |
| 11(R)-HPETE/Leukotriene B4 (isobars) | 225 |
| Prostaglandin A2 | 226 |
| Prostaglandin D2/E2/Thromboxane A2 (isobars) | 227 |
| Prostaglandin F2alpha/beta/D1 (isobars) | 228 |
| Prostaglandin D3 (isobars) | 229 |
| Prostaglandin G2 | 230 |
| 2-3-Dinor-8-iso prostaglandin F2alpha | 231 |
| Thromboxane B2 | 232 |
| Leukotriene A4 | 233 |
| Leukotriene C4 | 234 |
| **Plasticizers** |  |
| Phthalate | 235 |
| 2-Ethylhexyl phthalate | 236 |
| Di-n-propylphthalate | 237 |
| Bis(2-ethylhexyl)phthalate | 238 |
| **Other/tentative assignments** |  |
| Acetyl phosphate | 239 |
| N6 Methyl-L-lysine | 248 |
| Muramic acid | 249 |
| Sodium glucuronate | 258 |
| D-Ribitol-5-phosphate | 261 |
| Succinyl sulfathiazole | 265 |
| D-Ribose-5-diphosphate | 266 |
| S-Acylglutathione | 271 |
| N-Amidino-L-aspartate | 276 |
| Threo-3-Hydroxy-L-aspartate | 277 |
| N-Acyl-D-glutamate | 278 |
| Protein glutamine | 279 |
| Hydroxyacetone phosphate | 280 |
| N-Acyl-D-aspartate | 281 |
| N5-Methyl-L-glutamine | 283 |
| N-Acetylmethionine | 284 |
| Trans-Homoaconitate | 289 |
| Oxalosuccinate | 290 |
| N-Carbamyl-L-glutamate | 291 |
| Acylglycerone phosphate | 295 |

**
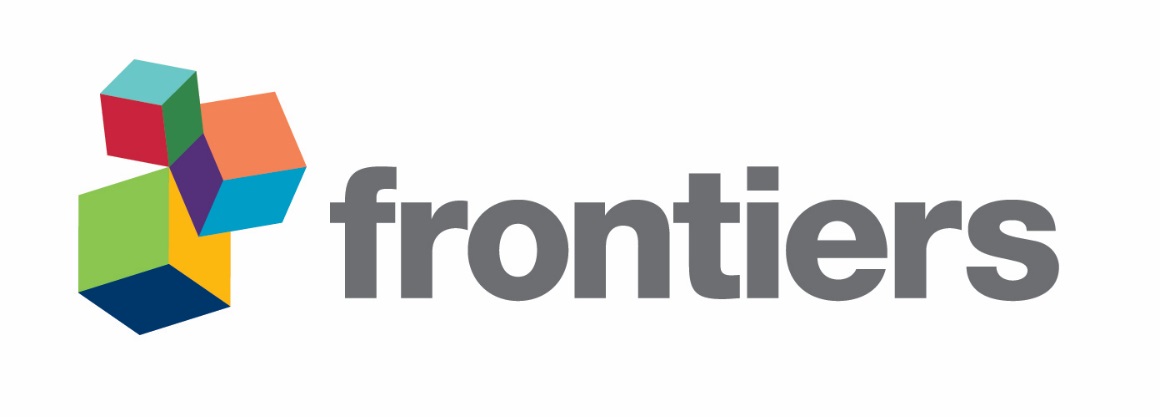
**
