# Supplementary material for: Metabolic Linkage and Correlations to Storage Capacity in Erythrocytes from Glucose 6-Phosphate Dehydrogenase-Deficient Donors
Source: Front Med (Lausanne). 2018 Jan 11;4:248. doi: 10.3389/fmed.2017.00248 (PMC5768619; doi:10.3389/fmed.2017.00248)
Supplement: Supplementary file 3 [file Table_3.docx]

Supplementary Material

**Metabolic linkage and correlations to storage capacity in erythrocytes from glucose 6-phosphate dehydrogenase deficient donors**

Julie A. Reisz^1†^, Vassilis L. Tzounakas^2†^, Travis Nemkov^1^, Artemis I. Voulgaridou^3^, Issidora S. Papassideri^2^, Anastasios G. Kriebardis^4*^, Angelo D’Alessandro^1*^, Marianna H. Antonelou^2^

^1^University of Colorado, School of Medicine,, Department of Biochemistry and Molecular Genetics, Anschutz Medical Campus, Aurora, CO, USA

^2^ National and Kapodistrian University of Athens, School of Science, Department of Biology, Athens, Greece

^3^“Apostle Paul” Educational Institution, Thessaloniki, Greece

^4^Technological and Educational Institute of Athens, Faculty of Health and Caring Professions, Department of Medical Laboratories, Athens, Greece

*** Correspondence:**Anastasios Kriebardis
[akrieb@biol.uoa.gr](mailto:akrieb@biol.uoa.gr)

Angelo D’Alessandro

[^ANGELO.DALESSANDRO@UCDENVER.EDU^](mailto:ANGELO.DALESSANDRO@UCDENVER.EDU)

^†^equal first authors

| **SUPPL. TABLE 3.** Point by point analysis of intraparameter correlations | | | | | | |
| --- | --- | --- | --- | --- | --- | --- |
| **NS** | **D7** | **D14** | **D21** | **D28** | **D35** | **D42** |
| Allantoate | 0.898* | 0.962** | 0.839* | 0.896* | 0.947** | 0.878* |
| Creatine | 0.942** | 0.837* | 0.967** | 0.818* | 0.877* | 0.879* |
| G6PD | N/D | N/D | N/D | N/D | N/D | 0.914* |
| Glycodeoxycholate | 0.833* | 0.829* | 0.844* | 0.843* | 0.843* | 0.829* |
| Guanidinoacetate | 0.946** | 0.892* | 0.818* | 0.873* | 0.830* | 0.889* |
| HbA1c | N/D | N/D | N/D | N/D | N/D | 0.978** |
| Hexadecanoic acid | 0.845* | 0.853* | 0.961** | 0.943** | 0.942** | 0.964** |
| iMCF | 0.941** | 0.970** | 0.992** | 0.953** | 1.000** | 0.928** |
| Indole | 0.824* | 0.943** | 0.834* | 1.000** | 0.943** | 0.943** |
| Lysine | 0.938** | 0.881* | 0.877* | 0.914* | 0.823* | 0.922** |
| MCF | 0.978** | 0.964** | 0.956** | 0.983** | 0.975** | 0.981** |
| MCH | N/D | 0.957** | N/D | 0.986** | N/D | 0.940** |
| MCV | N/D | 0.961** | N/D | 0.949** | N/D | 0.936** |
| NADPH | 0.962** | 0.907* | 0.813* | 0.826* | 0.847* | 0.833* |
| N-Succinyl-L-Citrulline | 0.969** | 0.924** | 0.841* | 0.865* | 0.980** | 0.829* |
| O-Propanoylcarnitine | 0.971** | 0.865* | 0.928** | 0.878* | 0.940** | 0.827* |
| Proline | 0.818* | 0.815* | 0.823* | 0.951* | 0.823* | 0.868* |
| Spermidine | 0.873* | 0.895* | 0.822* | 0.841* | 0.817* | 0.917** |
| TAC | 0.912* | 0.963** | 0.814* | 0.985** | 0.915** | 0.864* |
| UA-AC | 0.971** | 0.974** | 0.814* | 0.947** | 0.960** | 0.918** |
| Valine | 0.878* | 0.819* | 0.814* | 0.874* | 0.847* | 0.877* |
| (*), (**) Statistically significant Pearson or Spearman’s correlation coefficients *r* of non-stored (NS) vs. stored samples, *P* < .05 or *P* < .01 respectively. N/D, not determined. | | | | | | |

**
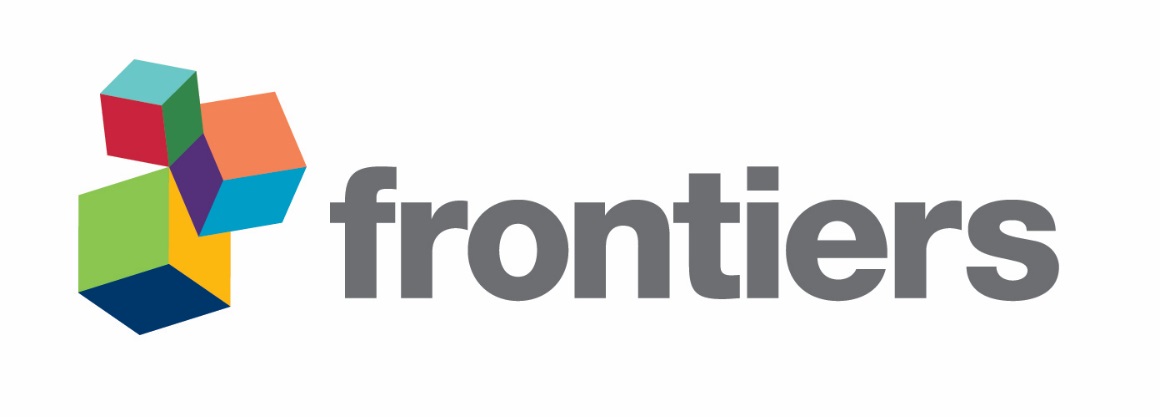
**
