# Supplementary material for: Metabolic Linkage and Correlations to Storage Capacity in Erythrocytes from Glucose 6-Phosphate Dehydrogenase-Deficient Donors
Source: Front Med (Lausanne). 2018 Jan 11;4:248. doi: 10.3389/fmed.2017.00248 (PMC5768619; doi:10.3389/fmed.2017.00248)
Supplement: Supplementary file 6 [file Image_3.PDF]

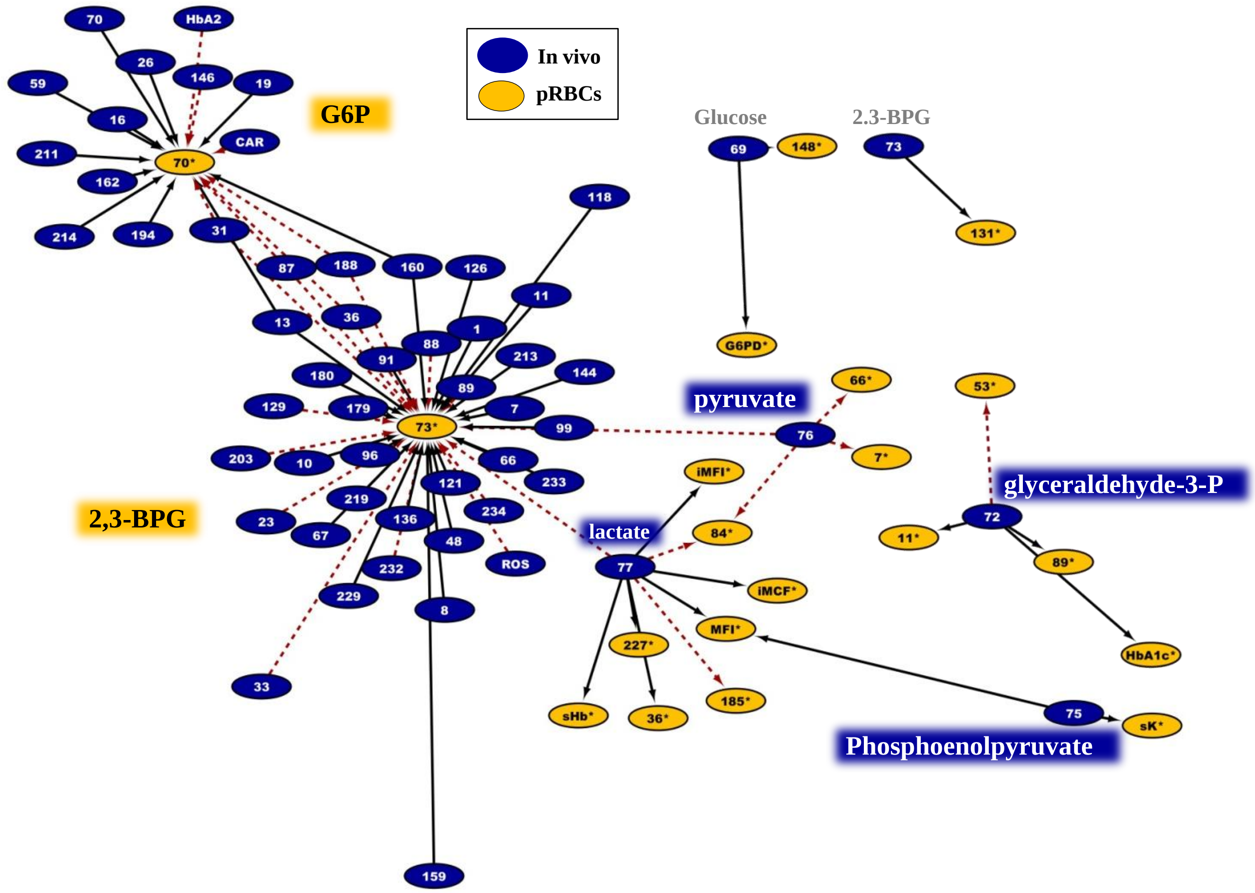

Supplementary Figure 3. Network presentation of *in vivo vs ex vivo* correlations among hematological, biochemical, physiological variables and metabolites of the glycolysis pathway in G6PD deficiency. Distinct hub nodes corresponding to in-bag levels of glucose-6-phosphate (G6P) and 2,3-BPG, as well as donor levels of lactate, G3P, pyruvate and phosphoenolopyruvate can be seen. The end-products of glycolysis *in vivo* had negative correlations with the levels of 2,3-BPG but opposite correlations with numerous hemolysis/fragility parameters of the RBC concentrates and supernatant K^+. In-bag levels of 2,3-BPG and glucose-6-phosphate represent the “target” of multiple factors in fresh blood, including both positive (amino acids, reduced glutathione-GSH, PPP, glycerophospholipids-GPLs, prostaglandin) and inverse (intracellular ROS and protein carbonylation, AMP/UTP, malate, leukotriene) correlations. Continuous black lines: positive correlations; Dashed red lines: negative correlations.
