# Supplementary material for: Metabolic Linkage and Correlations to Storage Capacity in Erythrocytes from Glucose 6-Phosphate Dehydrogenase-Deficient Donors
Source: Front Med (Lausanne). 2018 Jan 11;4:248. doi: 10.3389/fmed.2017.00248 (PMC5768619; doi:10.3389/fmed.2017.00248)
Supplement: Supplementary file 7 [file Image_4.PDF]

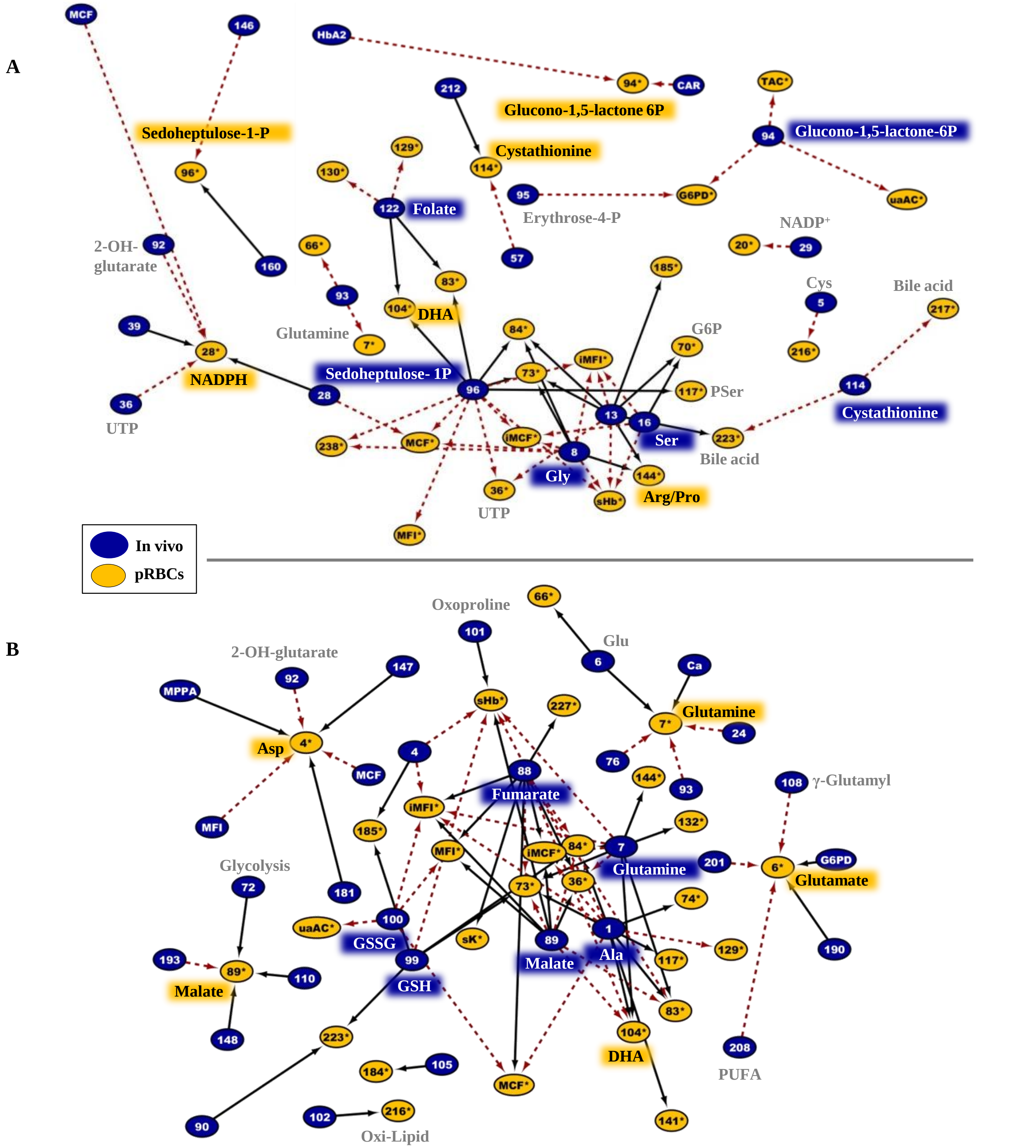

Supplementary Figure 4. Pentose phosphate (A) and GSH (B) pathways presentation by network analysis. (A) In the PPP sub-network the central role of the pre-storage levels of aminoacids, glucono-1,5-lactone-6P, sedoheptulose-1P and folate is obvious. (B) In the network involving GSH, malate and transaminases pathways, the metabolites malate, fumarate and glutamine had the highest degree of connectivity. In the PPP and one carbon metabolism pathways (**A**), the levels of critical metabolites in fresh RBCs (including amino acids, sedoheptulose-1-phosphate and folate) had positive correlations with in-bag levels of 2,3-BPG, PPP metabolites and redox variables (DHA) but inverse correlations with hemolysis-related metrics, such as extracellular Hb and susceptibility to osmotic or mechanical hemolysis. An interesting link between *in vivo* levels of protein carbonylation and osmotic fragility with the reducing power of the packed RBCs (NADPH, glucono-1,5-lactone, respectively) was also noticed. In reverse, *in vivo* levels of glucono-1,5-lactone-6P had strong correlations with those of the UA-dependent (and total) antioxidant capacity of the supernatant and of the G6PD activity. In the glutathione cycle (B), transaminases and malate-aspartate shuttle, the majority of connections concerned *in vivo* GSH/GSSG content, fumarate, glutamine, malate and alanine towards stored RBCs' amino acid and fatty acids metabolism, glutamine/glutamate, DHA, malate, 2,3-BPG, hemolysis and extracellular K^{+}. Continuous black lines: positive correlations; Dashed red lines: negative correlations.
