# Supplementary material for: Metabolic Linkage and Correlations to Storage Capacity in Erythrocytes from Glucose 6-Phosphate Dehydrogenase-Deficient Donors
Source: Front Med (Lausanne). 2018 Jan 11;4:248. doi: 10.3389/fmed.2017.00248 (PMC5768619; doi:10.3389/fmed.2017.00248)
Supplement: Supplementary file 10 [file Image_7.PDF]

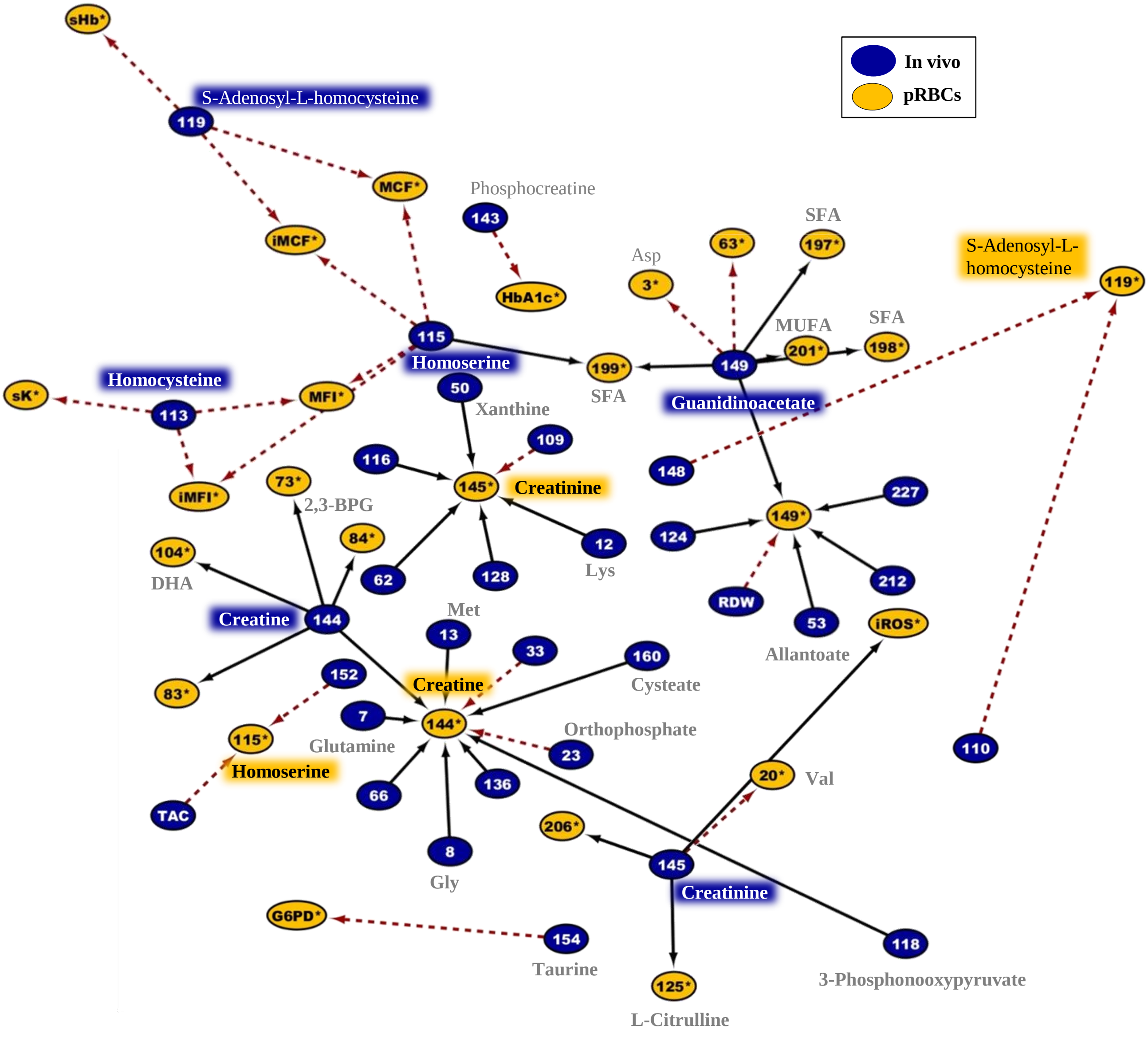

Supplementary Figure 7. The sulfur- and arginine metabolism-related part of the G6PD^{-}-specific *in vivo-vs- ex vivo* network shown in Supplementary Figure 2. Same as all networks currently reported, only Day 42 correlations are presented here, however, those pairs of statistically significant connections (with slightly different *r*) applied to all possible storage durations (Days 7, 14, 21, 28, 35 and 42). The length of each line is inversely proportional to the *r* value of the correlation (the shorter the edge, the higher *r* value). Continuous black lines: positive correlation; Dashed red lines: negative correlation.
