# Supplementary material for: Metabolic Linkage and Correlations to Storage Capacity in Erythrocytes from Glucose 6-Phosphate Dehydrogenase-Deficient Donors
Source: Front Med (Lausanne). 2018 Jan 11;4:248. doi: 10.3389/fmed.2017.00248 (PMC5768619; doi:10.3389/fmed.2017.00248)

Supplementary Figure 1. Extended vectorial overview of the hierarchical clustering analysis presented in Supplementary Table 1.

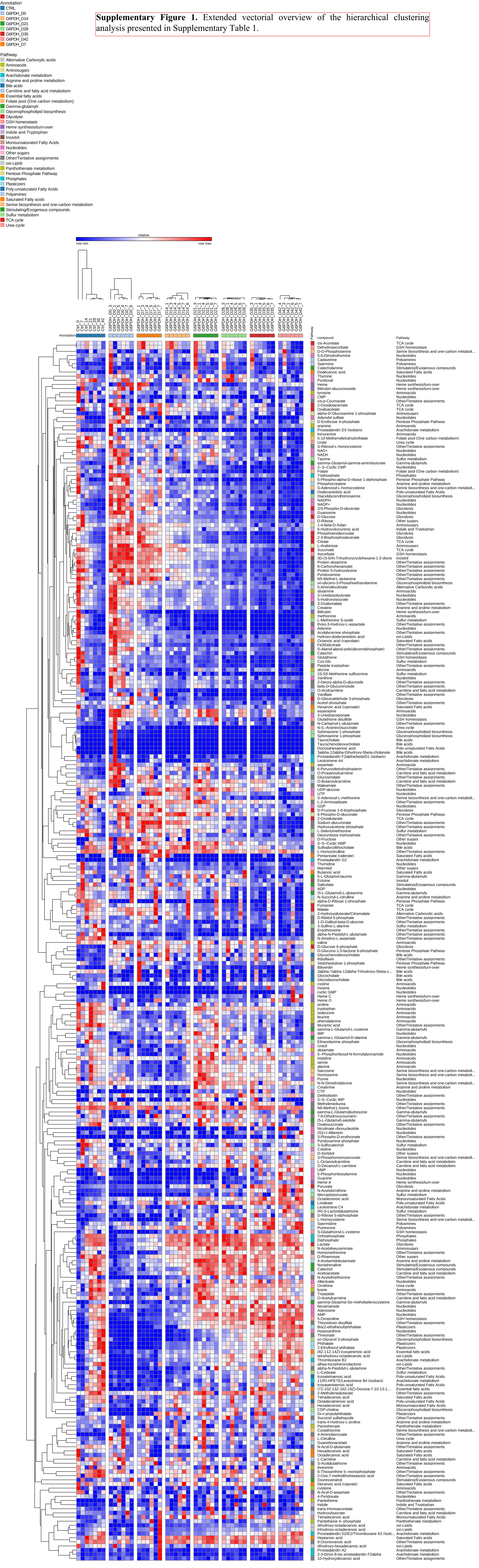

Supplement: Supplementary file 4 [file Image_1.PDF]
