# Supplementary material for: Metabolic Linkage and Correlations to Storage Capacity in Erythrocytes from Glucose 6-Phosphate Dehydrogenase-Deficient Donors
Source: Front Med (Lausanne). 2018 Jan 11;4:248. doi: 10.3389/fmed.2017.00248 (PMC5768619; doi:10.3389/fmed.2017.00248)
Supplement: Supplementary file 8 [file Image_5.PDF]

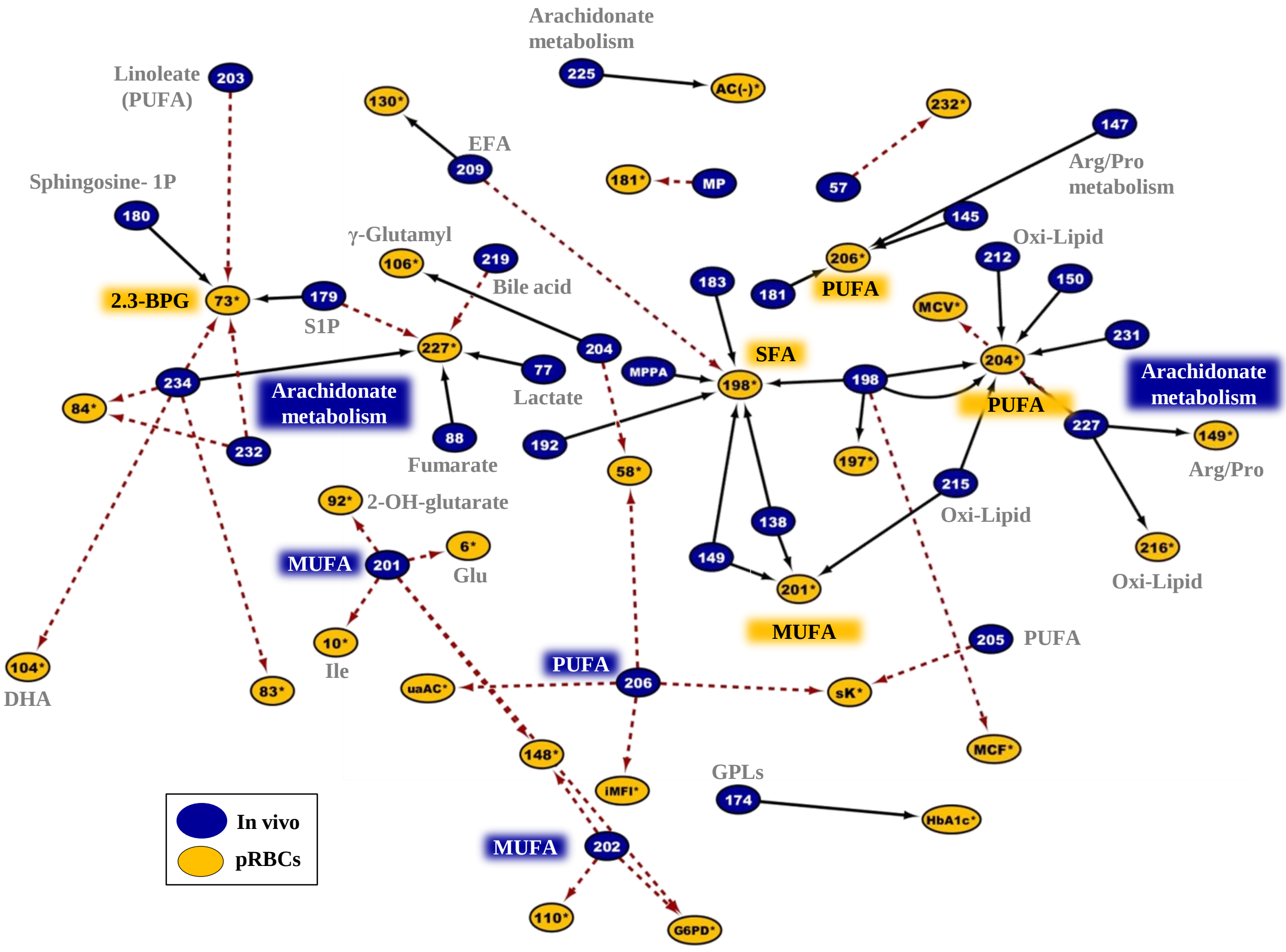

Supplementary Figure 5. Fatty acids-related interactome. EFA: essential fatty acids; MUFA: monounsaturated fatty acids; PUFA: polyunsaturated fatty acids. The fatty acids sub-network contained as much as 60 *in vivo/ex vivo* correlations. The levels of oxidized lipids, N-acetylornithine and carnitine at donation time had positive correlations with in-bag levels of the PUFA and MUFA, while the PUFA linoleate and members of the arachidonate metabolism (thromboxane B2, leukotriene C4), in opposite to the GPL sphingosine-1-phosphate, had inverse correlations with those of 2,3-BPG and DHA in the RBC unit. Of note, levels of eicosapentaenoic/eicosatetraenoic and hexadecanoic acids at donation correlated well (negatively) with the fragility of stored RBCs and the concentration of extracellular potassium in the supernatant. Another negative correlation connected linoleate concentration *in vivo* with that of stored RBCs' 2,3-BPG. Continuous black lines: positive correlations; Dashed red lines: negative correlations.
